# Therapeutic potential of stem cell extracellular vesicles for ischemic stroke in preclinical rodent models: a meta-analysis

**DOI:** 10.1186/s13287-023-03270-2

**Published:** 2023-04-03

**Authors:** Jing Zhao, Huiyin Deng, Chengfeng Xun, Chunli Chen, Zhiping Hu, Lite Ge, Zheng Jiang

**Affiliations:** 1 Department of Neurology, Second Xiangya Hospital, Central South University, Changsha, 410011 People’s Republic of China; 2 Hunan Provincial Key Laboratory of Neurorestoratology, The Second Affiliated Hospital, Hunan Normal University, Changsha, 410003 People’s Republic of China; 3 The National & Local Joint Engineering Laboratory of Animal Peptide Drug Development, College of Life Sciences, Hunan Normal University, Changsha, 410006 People’s Republic of China; 4 Department of Anesthesiology, The Third Xiangya Hospital, Central South University, Changsha, 410013 Hunan Province People’s Republic of China

**Keywords:** Exosomes, Brain, Stem cells, Extracellular vesicles, Ischemic stroke, Meta-analysis, Systematic review

## Abstract

**Background:**

Extracellular vesicles derived from stem cells (SC-EVs) have been proposed as a novel therapy for ischemic stroke. However, their effects remain incompletely understood. Therefore, we conducted this meta-analysis to systematically review the efficacy of SC-EVs on ischemic stroke in preclinical rodent models.

**Methods:**

Using PubMed, EMBASE, and the Web of Science, we searched through studies published up to August 2021 that investigated the treatment effects of SC-EVs in a rodent ischemic stroke model. Infarct volume was the primary outcome. Neurological severity scores (mNSS) were the secondary outcome. The standard mean difference (SMD) and the confidence interval (CI) were calculated using a random-effects model. R and Stata 15.1 were used to conduct the meta-analysis.

**Results:**

Twenty-one studies published from 2015 to 2021 met the inclusion criteria. We also found that SCs-EVs reduced infarct volume by an SMD of − 2.05 (95% CI − 2.70, − 1.40; *P* < 0.001). Meanwhile, our results revealed an overall positive effect of SCs-derived EVs on the mNSS with an SMD of − 1.42 (95% CI − 1.75, − 1.08; *P* < 0.001). Significant heterogeneity among studies was observed. Further stratified and sensitivity analyses did not identify the source of heterogeneity.

**Conclusion:**

The present meta-analysis confirmed that SC-EV therapy could improve neuron function and reduce infarct volume in a preclinical rodent ischemic stroke model, providing helpful clues for human clinical trials on SC-EVs.

**Supplementary Information:**

The online version contains supplementary material available at 10.1186/s13287-023-03270-2.

## Introduction

Stroke is one of the leading causes of death and disability among adults around the world [1]. Of all neurological disorders, stroke is one of the most common and devastating, accounting for 44 million physical disabilities, and 5.5 million deaths in the world yearly [2]. Because of the increasing prevalence, mortality, physical impairments, and ultimately the financial impact of stroke injuries, stroke injuries continue to contribute to problems for individuals and society [3]. The study of drugs and therapeutic practices for acute ischemic stroke has advanced in recent years [4]. Several early successes from preclinical studies have been translated into clinical trials, but the results are disappointing. Thus, it would be beneficial to examine more qualified strategies for stroke treatment.

As a type of cell with the potential for growing and self-renewing, stem cells (SCs), which include embryonic stem cells (ESCs), somatic stem cells, and iPSCs, are ideal for replacing damaged neural tissue and enhancing neurological function. The potential of stem cell therapy to treat ischemic stroke is great, with several clinical trials in progress [5]. Research conducted over the past decade has shown that stem cells are capable of treating a wide range of central nervous system diseases, including ischemic stroke [6]. Approximately 70 clinical trials have been conducted or are ongoing in relation to these diseases (ClinicalTrials.gov) [7]. Unfortunately, cell therapy has been found to be ineffective during clinical trials and preclinical studies primarily due to massive entrapment into the lung following intravenous administration [8, 9]. Additionally, injection of exogenous cells, although generally considered safe, can result in a malignant transformation [10].

In recent years, SC-EVs have emerged as a potential solution for nerve repair. A recent study showed that treatment of ischemic stroke patients with MSCs significantly increases circulating EVs, suggests the therapeutic role of MSC-derived EVs, and provides a mechanistic context for clinical findings of the trial [11]. On the other hand, various animal models have been widely used to study SC-EVs for the treatment of ischemic stroke. Extracellular vesicles (EVs) are small vesicles (of nanoscale) enclosing a lipid bilayer that contains genetic material (e.g., miRNAs, LncRNAs, etc.), proteins, small molecules, and lipids. Their characteristics differ depending on the parent cellular organelle [12]. Comparing EVs with polymeric or lipid-based nanoparticles, they offer a number of additional advantages including lower toxicity, immunogenicity, and the ability to cross biological barriers, such as the blood–brain barrier [9]. In general, SCs are believed to have therapeutic effects by way of paracrine mechanisms, including EVs [13]. The fact is that although EVs were at first considered merely as a means for cells to discard waste products, recently they have been attributed a wide range of roles in biological and pathological processes and even as therapeutics [14]. It has been reported that SC-EVs can achieve similar therapeutic effects to those of SCs, and they are considered safer than their parent cells [15]. Consequently, more and more studies have examined the use of EVs derived from SCs, specifically in the treatment of neuropathological disorders.

A systematic review consists of gathering, selecting, analyzing, and synthesizing all the relevant evidence to address a particular research question [16, 17]. Compared to traditional reviews, systematic reviews based on scientific methods provide a more objective evaluation of all the current relevant research evidence. This is a more accurate assessment of its findings, which is the highest level of scientific evidence quality [18, 19]. Although several animal studies have been conducted on various types of SC-EVs during early clinical trials, research-based evidence is still lacking in this area. To provide the most recent evidence regarding the efficacy of EVs in preclinical rodent models, we performed this meta-analysis. Additionally, our study will investigate the possible mechanisms by which the transplantation of SC-EVs can improve cognitive and behavioral deficits in animal models of ischemic stroke, potentially laying the foundations for the application of SC-EVs to patients who have suffered an ischemic stroke.

## Materials and methods

### Search Strategy

We searched the literature from the following databases: PubMed, Embase, Web of Science (until Aug 2021). Our search terms were as follows: (“mesenchymal stem cells” OR “mesenchymal stromal cells” OR “mesenchymal stem cell” OR “mesenchymal stromal cell” OR “Extracellular Vesicles” OR “Exovesicles” OR “Exosomes” OR “Endosomes”) AND (“Ischemic Strokes” OR “Infarct, Cerebral” OR “Cerebral Ischemia” OR “Stroke”) (The detailed search strategy is presented in Additional file 1.)

### Inclusion and Exclusion Criteria

The inclusion criteria for the analysis were as follows: (A) Experimental animals including mice, rats, and rodents; the following studies met the inclusion criteria. (B) The findings should be written and presented in English. (C) A preclinical rodent ischemic stroke model was induced. (D) They evaluated the efficacy of SC-EVs treatment in animal models of ischemic stroke (all types of animals of both sexes). (E) The studies provided adequate information regarding the neuron function and infarct volume. (F) It is imperative that the SC-EVs meet the standards of international guidelines for investigating EVs, which were published in 2018 and are entitled "Minimum Information for Studies of EVs" (MISEV 2018) [20]. (G) Report experimental results in original scientific publications. In the case of two or more articles with overlapping information, we select the most recent or most informative of the two.

Exclusion criteria were: (A) non-animal-based studies, in silico or in vitro. (B) Failure to provide information regarding the animal groups. (C) Study groups, without SC-EVs or those where the SC-EVs were not administered directly to animals. (D) Studies published more than once, duplicate reports, and abstracts without the complete text. (E) Literature reviews, organizational guidelines, letters, expert opinions, conference abstracts, or editorial correspondence without original data.(F) articles lacking significance and credibility.

### Study Selection

The records were managed by Endnote X9. Before performing any literature research, data were imported into Endnote X9. Next, duplicate records were identified and eliminated. Two researchers independently conducted the literature review based on the inclusion and exclusion criteria. Article titles and abstracts were initially screened to eliminate irrelevant articles. In addition, the remaining articles were assessed by obtaining the full text to identify the final articles included in the review. When there was a disagreement, another researcher was consulted.

### Data extraction

The data extraction procedure followed a detailed form that included the following information: Name of the author, year of publication, country, experimental methods (number of animals per group for individual comparisons), species, strain, and sex; methods of ischemic stroke induction in the animal model, sources and types of MSCs, the amount of SC-EVs, method of delivering SC-EVs, unit of dosage for SC-EV transplantation, time of administration, follow-up period, and clinical results. Two independent authors extracted data from the included studies. By using GetData Graph Digitizer software, values could be derived from images if only graphs were available. For instances in which the standard deviation was not available, we calculated the standard error by multiplying the SE by the square root of the group size. If results of various follow-ups or periods were evaluated at different times, only the longest period of follow-up was extracted. In addition, several independent groups (e.g., different EV doses, different delivery routes, and timings) were treated as separate datasets in a study.

### Risk of bias

The Systematic Review Centre for Laboratory animal Experimentation (SYRCLE) risk-of-bias tool was used by three independent reviewers to assess the potential for bias in each study included in the review. SYRCLE assesses selection bias, performance bias, detection bias, attribution bias, and reporting bias, reporting them as high, low, or unclear. Any disagreements were resolved following discussions with additional authors.

### Statistical Analysis

Statistical analysis was performed using the Stata 15.1 (StataCorp, College Station, TX, USA) [21], R language (version 4.1.3, www.r-project.org), and the meta-package (version 5.2–0) statistical software. The primary outcomes used for the analysis were Neurological severity scores (mNSS). The secondary outcome was the Infarct volume. To display the pooled mean difference, we generated forest plots based on the SMD and 95% confidence interval of each study. A difference of *P* < 0.05 is considered significant between the treatment and control groups. Heterogeneity was assessed based on I-squared (*I*_2_). The fixed-effects model was used to combine effect sizes for *I*_2_ > 50%, and the random-effects model for *I*_2_ ≤ 50%. We conducted subgroup analyses to identify potential sources of heterogeneity among the included studies. A sensitivity analysis examined overall stability. Egger's test was used if 10 or more datasets were included to check for potential publication bias, and the trim-and-fill method was also applied to data with publication bias.

## Results

### Identified and eligible studies

**Fig. 1:**
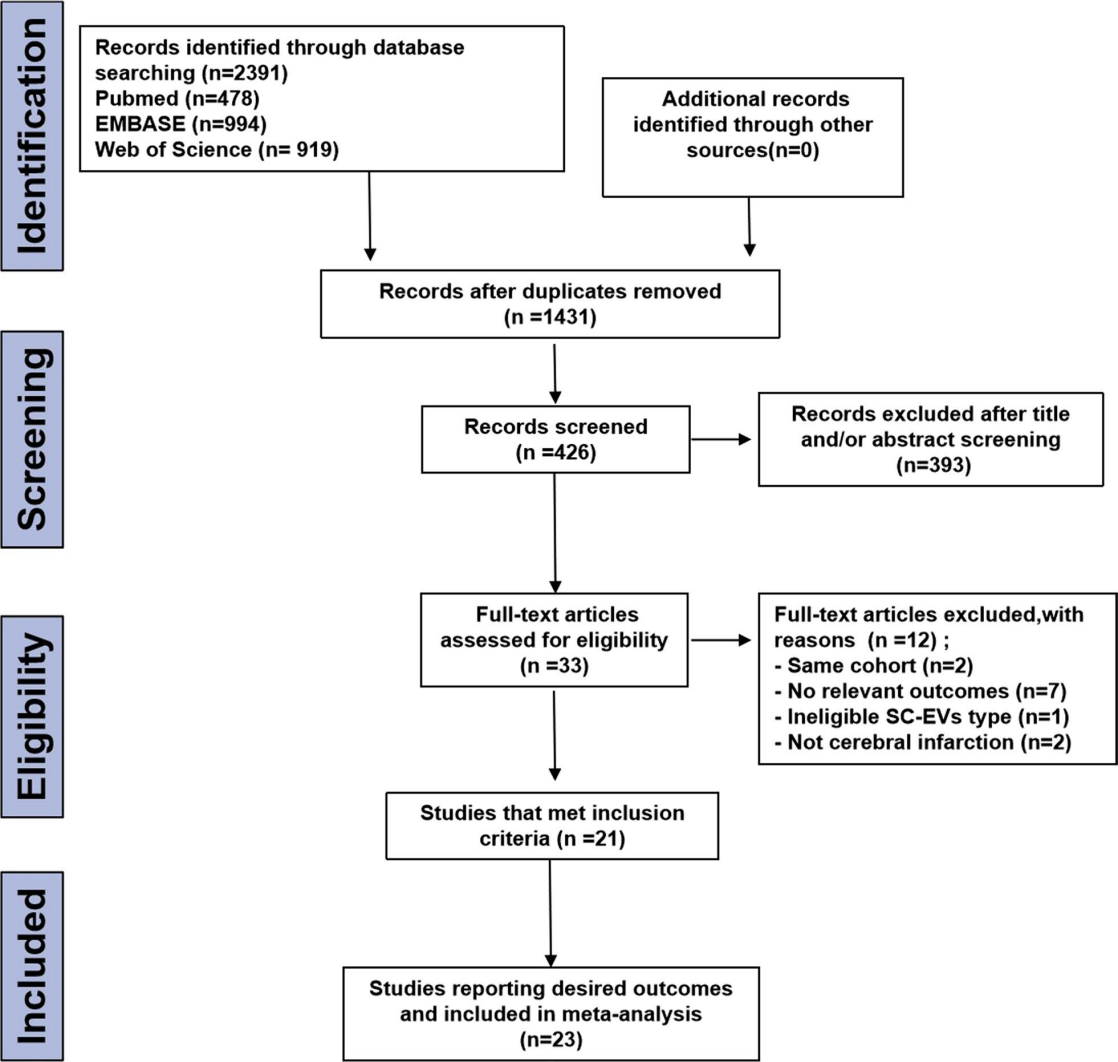
PRISMA flow diagram for review and selection process of studies included in meta-analysis of SC-EVs in rodent models of ischemic stroke

There were 2391 potential studies found in the primary retrieval: 478 in PubMed, 994 in Embase, and 919 in Web of Science. Among the 426 full-text articles remaining after review and exclusion, 33 were determined to be eligible for inclusion. 21 records of these were excluded as a result of the reasons indicated in Fig. 1. As a result of the meta-analysis, data from 21 studies (23 outcomes) published by 2021 were used. Out of the 21 studies, 18 reported infarct volume outcomes, 13 reported modified neurological severity scores (mNSS).

### Study characteristics

A total of 9 studies were conducted on rats and 12 on mice, 20 of which used the middle cerebral artery occlusion model (MCAO) to induce ischemic stroke, and the other study used the photothrombotic model of ischemic stroke. SC-EVs were obtained from xenograft in 10 studies and from allograft in 13 studies. Among the studies that used SC-EVs, 18 investigated MSCs, one investigated NSCs, one examined ESCs, and one examined induced pluripotent stem cells (iPS). As for MSCs, including adipose tissue-derived stem cells (ADSCs) 5 studies, bone marrow mesenchymal stem cells (BMSCs) 9 studies, dental pulp stem cells (DPSCs) 1 studies, placenta mesenchymal stem cells (PMSCs) 1 studies, and umbilical cord mesenchymal stem cells (UCMSCs) 2 studies. EV separation is generally accomplished through ultracentrifugation (*N* = 18), although polyethylene glycol (PEG) precipitation (*N* = 3) may also be employed. For most studies, SC-EVs were characterized by quantification, size distribution, morphological analysis, or expression of surface markers. The route of SC-EVs administration was intravenous in 14 studies, intracranial in 6 studies, and intra-arterial in 1 study. MSC-EVs were dosed in a wide variety of units, including absolute protein amount (*N* = 11), particle number (*N* = 6), and dosed by weight of the animal (*N* = 4). The majority of the studies involved a single transplant, and only two studies involved two to three transplants. Additionally, SC-EVs were given from 0 to 5 days following MCAO, with follow-ups ranging from 1 to 84 days. The characteristics of the included articles are summarized in Table 1 [22–42].

**Table 1:** Summary of study characteristics of all included articles

Number	Title	Author year Country	Species	Strain	Sex	Model	Stem cell type	Stem cell source	Compatibility SC-EVs Dose	SC-EVs dose units	Extraction method	Time of delivery	SC-EVs route	Follow-up
1	Microvesicles from brain-extract-treated mesenchymal stem cells improve neurological functions in a rat model of ischemic stroke	Lee et al. (2015) Republic of Korea [22]	Rats	Sprague–Dawley	Male	MCAO	MSC	Adipose tissues, Xenogenic	0.2 mg/kg	Dosed by weight of animal	DC	48 h after pMCAO	IA	7 days
2	MiR-17–92 cluster in exosomes enhance neuroplasticity and functional recovery after stroke in rats	Xin et al. (2018) USA [23]	Rats	Wistar	Male	MCAO	MSC	Bone marrow, Allogeneic	100 μg total exosome protein into 0.5 ml PBS	Protein amount	DC	24 h after MCAO	IV	28 days
3	Adipose-derived mesenchymal stem cells reduce autophagy in stroke mice by extracellular vesicle transfer of miR-25	Kuang et al. (2019) Germany [24]	Mice	C57BL/6	Male	MCAO	MSC	Adipose tissue, Allogeneic	200 μl of PBS corresponding to 10 μg of EVs	Protein amount	PEG	12 h after tMCAO	IV	14 days
4	In vivo Monitoring and Assessment of Exogenous Mesenchymal Stem Cell-Derived Exosomes in Mice with Ischemic Stroke by Molecular Imaging	Xu et al. (2020) China [25]	Mice	C57BL/6	Male	A photothrombotic model of ischemic stroke	MSC	Bone marrow, Allogeneic	100 μg, 200 μL PBS	Protein amount	DC	0 h	IV	14 days
5	miR-132-3p priming enhances the effects of mesenchymal stromal cell-derived exosomes on ameliorating brain ischemic injury	Pan et al. (2020) China [26]	Mice	C57BL/6	Not reported	MCAO	MSC	Bone marrow, Allogeneic	1 × 10^10 particles/100 μL in PBS	Particle number	DC	48 h after tMCAO	IV	2 days
6	Exosomes Derived from CXCR4-Overexpressing BMSC Promoted Activation of Microvascular Endothelial Cells in Cerebral Ischemia/Reperfusion Injury	Li et al. (2020) China [27]	Rats	SD	Male	MCAO	MSC	Bone marrow, Allogeneic	100 μg	Protein amount	DC	24 h after MCAO	ICV	21 days
7	Exosomes treatment mitigates ischemic brain damage but does not improve post-stroke neurological outcome	Nalamolu et al. (2020) USA [28]	Rats	Sprague–Dawley	Male	MCAO	MSC	Human umbilical cord blood, Xenogenic	150 μg/animal in 0.5 mL sterile PBS	Protein amount	DC	0 h	IV	7 days
8	Exosomes derived from human neural stem cells stimulated by interferon gamma improve therapeutic ability in ischemic stroke model	Zhang et al. (2020) China [29]	Rats	Sprague Dawley	Male	MCAO	NSC	Human fetal brain tissue, Xenogenic	4 × 10^9 particles in 10 ul PBS/each rat)	Particle number	DC	24 h after tMCAO	ICV	28 days
9	Small extracellular vesicles secreted by human iPSC-derived MSC enhance angiogenesis through inhibiting STAT3-dependent autophagy in ischemic stroke	Xia et al. (2020) China [30]	Rats	Sprague Dawley (SD)	Male	MCAO	IPS	ips, Xenogenic	1 × 10^11 particles in 500 μL PBS	Particle number	DC	4 h after tMCAO	IV	28 days
10	Lithium modulates miR-1906 levels of mesenchymal stem cell-derived extracellular vesicles contributing to post-stroke neuroprotection by toll-like receptor 4 regulation	Haupt et al. (2020) Germany [31]	Mice	C57BL/6	Male	MCAO	MSC	Bone marrow, Allogeneic	13.5 μg 1\3\5 day	Protein amount	PEG	1\3\5 day after MCAO	IV	84 days
11	Mesenchymal Stromal Cell-Derived Small Extracellular Vesicles Induce Ischemic Neuroprotection by Modulating Leukocytes and Specifically Neutrophils	Wang et al. (2020) Germany [32]	Mice	C57BL6/j	Male	MCAO	MSC	Bone marrow, Xenogenic	200 μL	Protein amount	PEG	0 h	IV	3 days
12	Bone mesenchymal stem cell-derived exosomal microRNA-29b-3p prevents hypoxic–ischemic injury in rat brain by activating the PTEN-mediated Akt signaling pathway	Hou et al. (2020) China [33]	Rats	Sprague Dawley (SD)	Male	MCAO	MSC	Bone marrow, Allogeneic	100 μg/kg/day 3 days	Dosed by weight of animal	DC	2 h MCAO	ICV	3 days
13	Human placental mesenchymal stem cells improve stroke outcomes via extracellular vesicles-mediated preservation of cerebral blood flow	Barzegar et al. (2021) USA [34]	Mice	C57Bl/6	Male	MCAO	MSC	Cholesterol treated placentas, Xenogenic	2 × 10^6 in 100 ml HBSS	Particle number	DC	1 h MCAO	IV	1 day
14	Human umbilical cord mesenchymal stem cell-derived exosomal miR-146a-5p reduces microglial-mediated neuroinflammation via suppression of the IRAK1/TRAF6 signaling pathway after ischemic stroke	Zhang et al. (2021) China [35]	Mice	C57BL/6	Not reported	MCAO	MSC	Umbilical cord mesenchymal stem cell, Xenogenic	250 μL PBS with 50 μg	Protein amount	DC	4 h after tMCAO	IV	3 days
15	Dental pulp stem cell-derived exosomes alleviate cerebral ischemia–reperfusion injury through suppressing inflammatory response	Li et al. (2021) China [36]	Mice	C57BL/6	Male	MCAO	MSC	Dental pulp, Xenogenic	10 μg total protein per 100 μL PBS	Protein amount	DC	2 h MCAO	ICV	7 days
16	Upregulation of extracellular vesicles-encapsulated miR-132 released from mesenchymal stem cells attenuates ischemic neuronal injury by Inhibiting smad2/c-jun pathway via acvr2b suppression	Feng et al. (2021) China [37]	Mice	C57BL/6 J	Male	MCAO	MSC	Bone marrow, Allogeneic	200 mg EVs	Protein amount	DC	24 h after tMCAO	IV	1 day
17	miR-31 from adipose stem cell-derived extracellular vesicles promotes recovery of neurological function after ischemic stroke by inhibiting TRAF6 and IRF5	Lv et al. (2021) China [38]	Mice	C57BL/6 J	Male	MCAO	MSC	Adipose tissue, Allogeneic	150 μg EVs	Protein amount	DC	24 h after tMCAO	IV	14 days
18	MiR-17–92 enriched exosomes derived from multipotent mesenchymal stromal cells enhance axon–myelin remodeling and motor electrophysiological recovery after stroke	Xin et al. (2021) USA [39]	Rats	Wistar	Male	MCAO	MSC	Bone marrow, Allogeneic	3 × 10^11 particles	Particle number	DC	24 h after MCAO	IV	28 days
19	Embryonic stem cell-derived small extracellular vesicles modulate regulatory T cells to protect against ischemic stroke	Xia et al. (2021) China [40]	Mice	C57/BL6	Male	MCAO	ESC	Human embryonic stem cells, Xenogenic	10^9 particles in 200 μL of PBS	Particle number	DC	0 h injected three times daily	IV	28 days
20	Exosomal microRNA-22-3p alleviates cerebral ischemic injury by modulating KDM6B/BMP2/BMF axis	Zhang et al. (2021) China [41]	Rats	Sprague Dawley (SD)	Male	MCAO	MSC	Adipose tissue, Allogeneic	100 μg/kg/day 3 days	Dosed by weight of animal	DC	Before the surgery	ICV	3 days
21	microRNA-26a shuttled by extracellular vesicles secreted from adipose-derived mesenchymal stem cells reduce neuronal damage through KLF9-mediated regulation of TRAF2/KLF2 axis	Hou et al. (2021) China [42]	Mice	C57BL/6	Male	MCAO	MSC	Adipose tissue, Allogeneic	100 mmol/kg/d,3 days	Dosed by weight of animal	DC	Before the surgery	ICV	3 days

### Risk of bias

To assess the study design and reporting of the study, we used the Systematic Review Centre for Laboratory animal Experimentation (SYRCLE) tool for identifying potential bias in animal preclinical studies [43]. In Table 2, the risk of bias is summarized across all included studies. Overall, no study was judged to have a low risk of bias. In most studies, the methods relating to sequence generation, allocation concealment, random housing, random outcome assessment, and blinding of assessors were not described in any detail. Moreover, several studies about the baseline characteristics of the animals, the blinding of the assessors, and the selective reporting of the outcomes have been described. However, studies evaluated for attrition bias varied in risk, with a high risk of bias assigned to 31.8% of the studies (*N* = 7), which failed to account for declines in animal numbers reported between methods and results; 45.4% of the studies (*N* = 10) were at low risk, and the remainder (*N* = 4) were unclear. A lack of published protocols made it impossible to determine whether selective reporting bias existed across almost all studies. Additional sources of bias were not identified.

**Table 2:** SYRCLE risk-of-bias assessment for included studies

Number	Author (year)	Author year country	Random sequence generation?	Groups similar at baseline?	Allocation concealed?	Animals randomly housed?	Blinding of caregivers and/or examiners?	Random selection for outcome assessment?	Blinding of outcome assessor?	Incomplete outcome data addressed?	Free from selective outcome reporting?	Free from other bias?
1	2015	Lee et al. (2015) Republic of Korea [22]	L	U	L	U	L	L	L	L	L	L
2	2019	Xin et al. (2018) USA [23]	U	U	U	U	L	U	L	L	L	L
3	2019	Kuang et al. (2019) Germany [24]	L	L	U	L	L	U	U	L	L	L
4	2020	Xu et al. (2020) China [25]	U	U	U	U	U	U	U	H	U	L
5	2020	Pan et al. (2020) China [26]	U	L	U	L	U	U	U	H	L	L
6	2020	Li et al. (2020) China [27]	U	U	U	U	U	U	U	H	U	L
7	2020	Nalamolu et al. (2020) USA [28]	L	L	L	L	L	L	L	L	L	L
8	2020	Zhang et al. (2020) China [29]	U	L	U	U	L	L	L	L	L	L
9	2020	Xia et al. (2020) China [30]	L	L	U	L	U	U	U	U	L	L
10	2020	Haupt et al. (2020) Germany [31]	L	L	U	U	L	U	U	U	L	L
11	2020	Wang et al. (2020) Germany [32]	U	U	U	U	U	U	U	H	L	L
12	2020	Hou et al. (2020) China [33]	L	L	U	L	L	U	L	L	L	L
13	2021	Barzegar et al. (2021) USA [34]	U	U	U	U	U	U	U	H	L	L
14	2021	Zhang et al. (2021) China [35]	U	L	U	U	L	L	L	H	L	L
15	2021	Li et al. (2021) China [36]	L	L	L	L	L	U	L	H	U	L
16	2021	Feng et al. (2021) China [37]	U	U	U	U	L	L	L	L	L	L
17	2021	Lv et al. (2021) China [38]	L	L	U	L	U	U	U	L	L	L
18	2021	Xin et al. (2021) USA [39]	U	U	U	U	L	U	L	L	L	L
19	2021	Xia et al. (2021) China [40]	L	L	U	L	U	U	U	L	L	L
20	2021	Zhang et al. (2021) China [41]	U	L	U	U	U	U	U	U	L	L
21	2021	Hou et al. (2021) China [42]	U	L	U	U	U	U	U	U	L	L

### Meta-analysis and effect evaluation

**Fig. 2:**
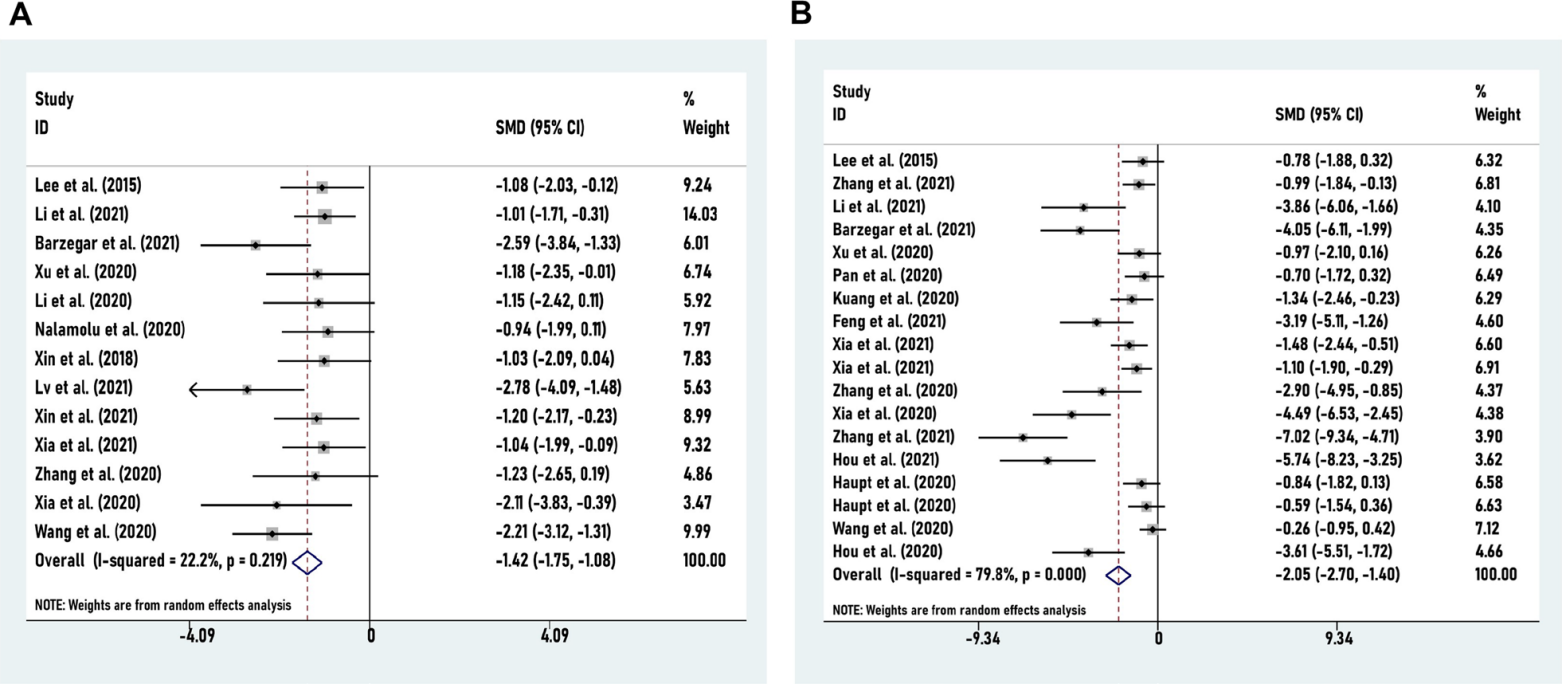
Forest plot shows the mean effect size and 95% confidence interval (CI) for mNSS (**A**) and infarct volume outcomes (**B**) between SC-EVs treatment group and control group in all studies

For ischemic stroke, SC-EVs administration led to favorable outcomes for the functional mNSS, as well as histopathological outcomes for infarct volume. Accordingly, the composite weighted mean of mNSS score (*N* = 13) was − 1.42 (95% CI: − 1.75 to − 1.08, *I*^2^ = 22.2%), (*P* < 0.001) (Fig. 2A), and infarct volume (*n* = 18) was − 2.05 (95% CI: − 2.70 to − 1.40, *I*^2^ = 79.8%), (*P* < 0.001) (Fig. 2B). Results of these studies have demonstrated that SC-EVs have a beneficial effect on ischemic stroke models. In accordance with the *I*^2^ statistic, comparisons of infarct volume outcomes are extremely heterogeneous (*P* = 0.000).

### Subgroup analysis

Further subgroup analyses were conducted on the infarct volume based on different categories, which are described in Additional file 1: Figure S1-8. Generally, SC-EVs were found to be effective in the majority of subgroups, but not in a few subgroups as a whole (*P* < 0.05). No differences in effect size were observed among immunocompatibility (allogeneic versus xenogeneic) (*P* = 0.48) (Additional file 1: Figure S1), species (*P* = 0.06) (Additional file 1: Figure S2), and ischemic stroke model (*P* = 0.08) (Additional file 1: Figure S3). However, sources of SCs (*P* < 0.01) (Additional file 1: Figure S4), species sex (*P* < 0.01) (Additional file 1: Figure S5), route of administration (*P* < 0.01) (Additional file 1: Figure S6) and timing of treatment (*P* < 0.01) (Additional file 1: Figure S7), and extraction method of SC-EVs (*P* < 0.01) (Additional file 1: Figure S8) may result in differential effects. Stratified analyses were able to reveal significant differences between groups, but the source of this heterogeneity was unable to be identified.

### Publication Bias

**Fig. 3:**
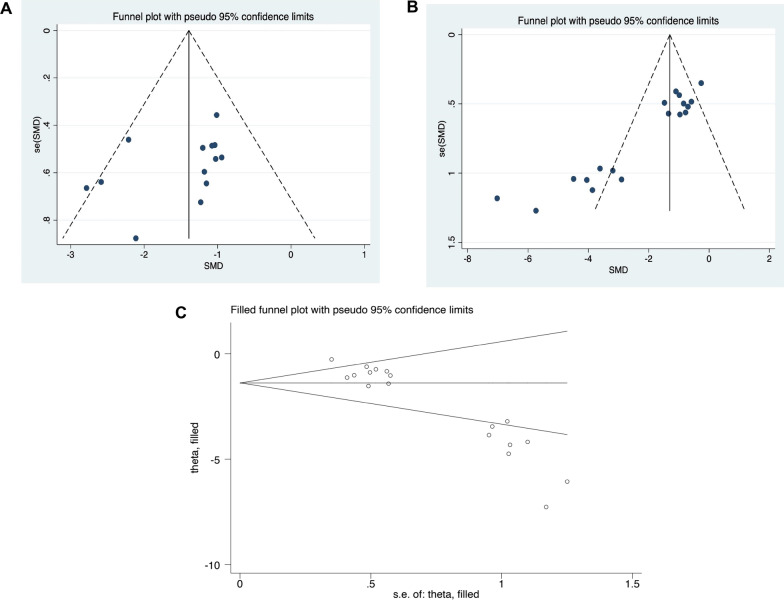
Evaluation of publication bias. Funnel plots for mNSS (**A**) and infarct volume outcomes (**B**), with the y-axis signifying study quality and the x-axis showing the study results. **C** Trim-and-fill method was used to evaluate the missing studies in infarct volume outcomes. SMD, standardized mean difference

According to Fig. 3A, B, the funnel plot for cerebral infarction showed asymmetry for comparisons of mNSS (*P* = 0.159) and infarct volume (*P* = 0.000). According to Egger's test, there is an obvious publication bias. Following this, we applied the trim-and-fill strategy to evaluate missing studies and recalculated the overall estimate of the pooled effect. The estimates of the imputed effect of infarct volume were comparable to the previous estimates (SMD: − 2,048, 95% CI: − 2.697 to − 1.399, *P* = 0.000), which clearly indicates no "missing" studies (Fig. 3C).

### Sensitivity analysis

**Fig. 4:**
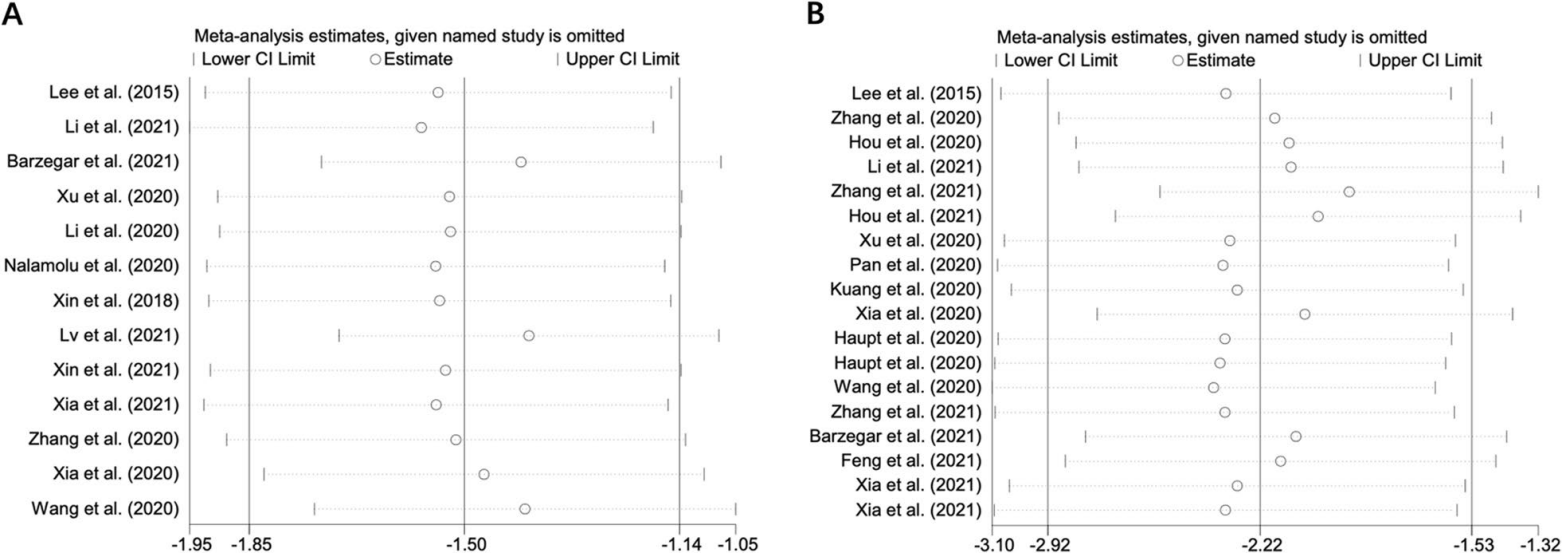
Sensitivity analysis of the studies included in mNSS (**A**) and infarct volume outcomes (**B**)

Considering the notable heterogeneity of the studies, we conducted a sensitivity analysis to assess the stability of results by sequentially omitting each study. The pooled SMD of mNSS and infarct volume outcomes did not differ among studies as shown in Fig. 4A, B.

## Discussion

### Main Findings

Our meta-analysis of 21 records provided a comprehensive summary of the impacts of SC-EV therapies on the rodent model of ischemic stroke after SC-EV treatments were administered. In preclinical rodent models of ischemic stroke, SCs-EVs were found to reduce infarct volume and improve neurological deficits in the analyses. Consequently, the current meta-analysis provides valuable information for human clinical trials using SC-EVs. Since the limited number of studies, it will take more evidence to prove the neuroprotective effect of SC-EVs treatments in experimental ischemic stroke.

### Possible mechanisms of SC-EVs for ischemic stroke

**Fig. 5:**
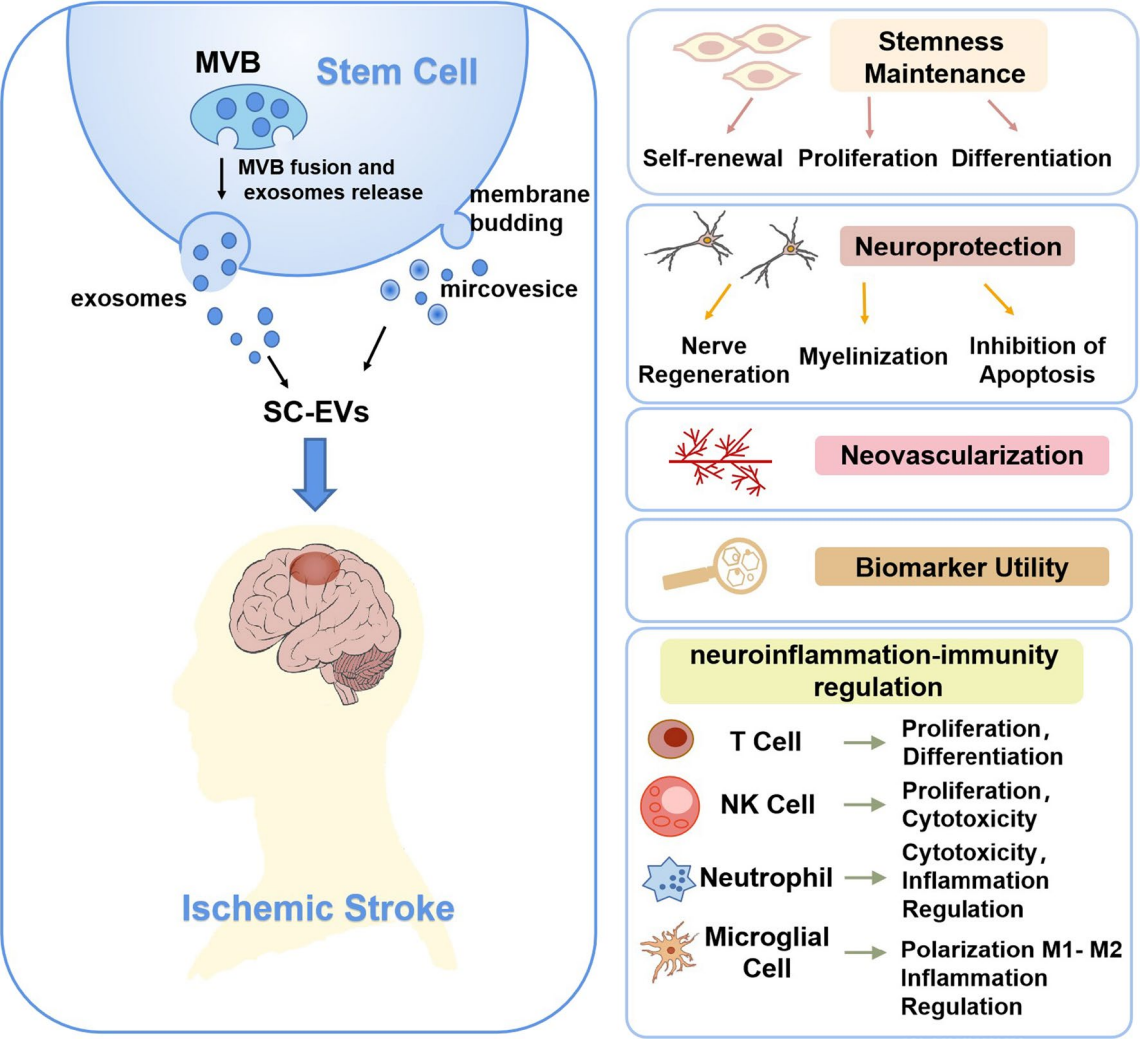
Possible mechanisms of SC-EVs therapy for ischemic stroke. Extracellular vesicles (EVs) mainly include exosomes and microvesicles. Exosomes originate from multivesicular bodies (MVB) and microvesicles are formed through cell membrane budding. SC-EVs can repair damaged brains and nerve tissues by maintaining stem cells, neuroprotection, angiogenesis, biomarker utility, and neuroinflammation–immunity regulation

Although a number of preclinical studies have demonstrated the potential for SC-EVs in regenerative medicine, detailed research on the mechanisms behind neurological functional recovery has yet to be conducted. Based on preclinical studies, SC-EVs appear to promote the repair of nerve tissue damage by maintaining stem cells, neuroprotection, angiogenesis, biomarker utility, and neuroinflammation–immunity regulation (Fig. 5). (a) Stemness maintenance. In order to regenerate tissue, endogenous stem cells need to proliferate, self-renew, and differentiate. It has been shown that SC-EV contains mRNAs encoding stem-associated transcription factors, such as Nanog, Oct4, HoxB4, and Rex-1, all of which are essential for maintaining stem cell characteristics [44]. Moreover, endogenous stem cells can be stimulated to proliferate, self-renew, or differentiate through the transfer of molecules such as Wnt3 [44], Hedgehog [45], as well as other molecules. (b) Neuroprotection. According to a study conducted by Zhang et al., injection of EVs that target miR-17–92 increased neurogenesis, oligodendrogenesis, and neural plasticity using intravenous injection of ischemic stroke model [35]. The molecular bases for these restorative changes may in-part be attributed to the miR-17–92 cluster down-regulation of PTEN expression and subsequent activation of PTEN downstream proteins, Akt, and mTOR, as well as inhibition of GSK-3β activity [23]. (c) Angiogenesis. Angiogenesis is a pathophysiological process associated with tissue regeneration and reconstruction. The transplantation of the SC-EVs can enhance angiogenesis in the tissue as demonstrated by the change in expression of VEGF after the transplantation [46]. EVs are believed to play an essential role in angiogenesis and revascularization of the cerebrovasculature, primarily through the secretion of angiogenic factors and noncoding RNAs, including microRNAs, long noncoding RNA, circular RNA, and miRNAs. During the injection of SC-EVs into animals, endogenous VEGF and VEGFR2 levels are increased in the ischemic zone [47, 48]. EVs derived from SCs carrying miR-125a, miR-21, and miR-612 were able to regulate expression of pro-angiogenic genes in vitro, including angiopoietin-1 (Ang1), fetal liver kinase-1 (Flk1), VEGF, and others [47, 48]. (d) Biomarker utility of SC-EVs. Despite the therapeutic potential, EVs could also serve as biomarkers for SCs therapy and other pathophysiological processes. Recent research conducted by Dr. Bang et al. found that ischemic stroke patients treated with mesenchymal stem cells had significant increases in extracellular vesicles, correlated with increased motor function and MRI plasticity measures [11]. (e) Neuroinflammation–immunity regulation. Previous studies have shown that SC-EVs significantly suppress the inflammatory response by regulating the polarization of microglia [49]. In addition, preclinical studies have demonstrated that SC-EVs can be used to modulate immune parameters in the treatment of various diseases through the delivery of noncoding RNAs, cytokines, and other immunomodulatory molecules. According to Xia et al., ESC-EVs contribute to the increase in regulatory T cells (Tregs) after stroke. By increasing the proportion of Treg cells, ESC-EVs modulate neuroinflammation, and thereby protect against ischemic stroke. This process is mediated by the activation of the TGF-β/Smad signaling pathway by the transfer of TGF-β, Smad2, and Smad4 [40].

### Prospects and clinical challenges of SC-EVs therapy for ischemic stroke

**Fig. 6:**
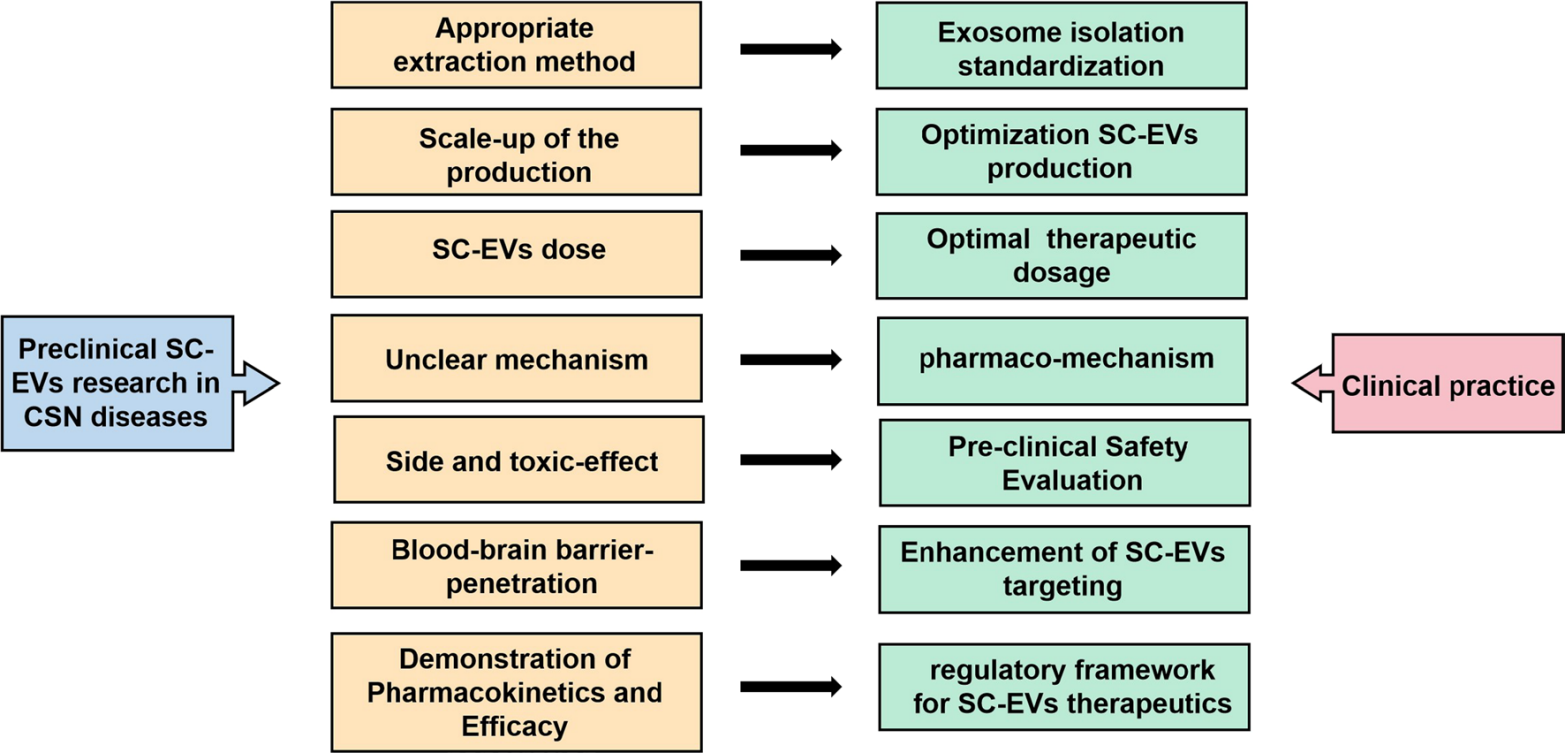
Clinical challenges of MSC-EVs therapy for ischemic stroke

The clinical potential of SCs has been increasingly studied over the past decade for various ischemic strokes. With the advancement of research, growth in recognition of and praise for the paracrine function of SCs has increased [5]. As the most significant part of paracrine, EVs have become a new research hotspot and are even being tested in clinical studies. Recently, Dr. Bang et al. [11] conducted the first randomized controlled trial involving 54 patients with ischemic stroke, indicating that circulating levels of EV were significantly higher after the injection of MSCs within 24 h, suggesting that EV has significant potential for treating cerebral infarction. Even though SC-EVs have generated a lot of interest as a promising therapy for ischemic stroke, there are many challenges that must still be addressed before fully exploiting the potential of EVs as a result of the youth of the field. Since animal models provide an important framework for designing clinical trials, it is important to examine the combined effects of preclinical studies. Meanwhile, further studies are required to evaluate the potential of MSC-derived EV therapeutics in stroke patients. The therapeutic applications of SC-EVs for numerous CNS diseases hold considerable promise; however, there are several challenges associated with their use (Fig. 6). (A) An initial consideration is the technical challenge, ranging from the isolation of EV to its characterization and standardization for clinical applications. In most studies, SC-EVs are typically isolated using low-throughput techniques such as ultrasound centrifugation. Therefore, advancing techniques and methods are needed, such as tangential flow filtration or size exclusion chromatography utilizing techniques, offering the prospect of preparing SC-EVs from large volumes of culture media. (B) Another significant technical challenge is scaling up SCs culture so as to produce enough SC-EVs for clinical use. The use of bioreactors and 3D stem cell culture may offer a viable solution to this problem [50]. (C) As a matter of fact, determining the effective dose of therapeutic SC-EVs, as well as the mode of action, remains a difficult task for the field. Throughout the studies, we found different units of dosage for SC-EVs, including absolute protein amounts, particle number, the amount of EVs released by a specific number of SCs, and EVs released continuously or dosed by the weight of the animal. The lack of uniformity of units is therefore detrimental to the development of new therapeutics for SC-EVs. To facilitate research into the optimal therapeutic dose, it is imperative that the unit is unified as soon as possible. (D) A further challenge in this area is determining the mechanisms of action of therapeutics containing SC-EVs. A deeper understanding of the SC-EVs mechanism of action will enable the development of appropriate dose and functional assessments. Due to this, once we gain a greater understanding of the therapeutic potential of SC-EVs, we may be able to optimize the extraction process to obtain higher levels of function from SC-EVs. (E) The appropriate source of stem cells for SC-EV isolation and therapeutic applications is also critical due to challenges relating to immunogenicity and to ensure that EVS derived from stem cells do not carry harmful epigenetic changes. This can be addressed by developing appropriate preclinical models and selecting formulations of SC-EV with desired molecular characteristics. It will also be helpful for the choice of these parameters to understand the mechanisms by which specific formulations of SC-EVs function in a therapeutic setting. (F) SC-EVs that have been demonstrated to cross the BBB have shown the ability to reach organs such as the brain. Further investigation is required to determine whether SC-EVs can be directed to the specific sites at which they will exert their therapeutic effect in the treatment area. Moreover, other methods of improving the targeting of SC-EVs might also be considered. (G) Furthermore, there is no regulatory framework for EV therapeutics, although they may belong to the pharmaceutical class of biologicals. Clinically approved therapeutic agents must demonstrate their pharmacokinetics and therapeutic efficacy, and these are currently in their infancy in the field of EVs. Although there are still technical and regulatory hurdles to overcome, as progressively more studies demonstrate, it is clear that SC-EVs have enormous potential for therapeutic applications.

### Strengths and limitations

To our knowledge, this is the first systematic review of animal studies assessing the therapeutic efficacy of SC-EVs in treating ischemic stroke. However, some limitations should be discussed. The first constraint is that we can only include studies that have already been published in English as part of our methodology. Unpublished data can influence our conclusions. Additionally, our study concluded that head-to-head comparisons of the EV methodology and/or subtypes of approaches should be conducted in order to identify the most efficient clinical translation strategies. In order for a study to be credible, it should utilize an adequate sample size and a formal calculation [51]. Meta-analyses are clearly affected by poor study quality and substantial publication bias, as well as low external and internal validity. Consequently, there is a certain degree of amplifying of the efficacy of SC-EVs therapy for ischemic stroke since studies that are left tend to confirm neutral or negative results. As a final note, although there were no adverse events reported, none of the studies included in the review conducted formal tests to investigate the safety of SC-EVs.

## Conclusions and future directions

Ischemic stroke has limited treatment options, which calls for novel approaches. Rodent studies have demonstrated that SC-EV is an effective treatment for ischemic stroke. We believe that our meta-analysis will serve as a valuable source of reference for future preclinical and clinical studies having important implications for human health. There are still differences, limitations, and irregularities in the routes, dosage, and dosage unit of SC-EV administration and the source of stem cells or time for transplantation therapy, among the studies included. To support further clinical translation, improvements must be made in study design, outcome measurement, and quality assurance to minimize bias and scientifically investigate the role of SC-EVs in ischemic stroke treatment. In addition, more evidence-based research should be conducted to strengthen the clinical translation of SC-EVs.

## Supplementary Information

**Additional file 1.** The Detailed Search Strategy and Subgroup analysis figures.

## Data Availability

The original contributions presented in the study are included in the article/Supplementary Material. Further inquiries can be directed to the corresponding author.

## Abbreviations

ADSCs: Adipose tissue-derived stem cells
Ang1: Angiopoietin-1
BMSCs: Bone marrow mesenchymal stem cells
CI: Confidence interval
DPSCs: Dental pulp stem cells
ESCs: Embryonic stem cells
EVs: Extracellular vesicles
Flk1: Fetal liver kinase-1
ICV: Intracerebroventricular
IV: Intravenous
MCAO: Middle cerebral artery occlusion model
mNSS: Neurological severity scores
PEG: Polyethylene glycol
PMSCs: Placenta mesenchymal stem cells
PRISMA: Preferred reporting items for systematic review and meta-analyses
SCs: Stem cells
SD: Standard deviation
SE: Standard error
SMD: Standardized mean difference
Tregs: Regulatory T cells
UCMSCs: Umbilical cord mesenchymal stem cells
