# Supplementary material for: Therapeutic potential of stem cell extracellular vesicles for ischemic stroke in preclinical rodent models: a meta-analysis
Source: Stem Cell Res Ther. 2023 Apr 3;14:62. doi: 10.1186/s13287-023-03270-2 (PMC10071642; doi:10.1186/s13287-023-03270-2)
Supplement: Supplementary file 1 — Additional file 1. The Detailed Search Strategy and Subgroup analysis figures. [file 13287_2023_3270_MOESM1_ESM.doc]

**Supplement [Materials](../../../../D:/%25E8%25BD%25AF%25E4%25BB%25B6/360%25E6%25B8%25B8%25E8%25A7%2588%25E5%2599%25A8/Youdao/Dict/8.9.9.0/resultui/html/index.html#/javascript:;)**

**Additional file 1**

**The Detailed Search Strategy**

**Pubmed**：

(Cerebral Infarctions[Title/Abstract]) OR (Infarctions, Cerebral[Title/Abstract]) OR (Infarction, Cerebral[Title/Abstract]) OR (Cerebral Infarct[Title/Abstract]) OR (Cerebral Infarcts[Title/Abstract]) OR (Infarct, Cerebral[Title/Abstract]) OR (Infarcts, Cerebral[Title/Abstract]) OR (Cerebral Infarction, Left Hemisphere[Title/Abstract]) OR (Left Hemisphere, Infarction, Cerebral[Title/Abstract]) OR (Infarction, Left Hemisphere, Cerebral[Title/Abstract]) OR (Left Hemisphere, Cerebral Infarction[Title/Abstract]) OR (Cerebral, Left Hemisphere, Infarction[Title/Abstract]) OR (Infarction, Cerebral, Left Hemisphere[Title/Abstract]) OR (Subcortical Infarction[Title/Abstract]) OR (Infarction, Subcortical[Title/Abstract]) OR (Infarctions, Subcortical[Title/Abstract]) OR (Subcortical Infarctions[Title/Abstract]) OR (Posterior Choroidal Artery Infarction[Title/Abstract]) OR (Anterior Choroidal Artery Infarction[Title/Abstract]) OR (Cerebral Infarction, Right Hemisphere[Title/Abstract]) OR (Right Hemisphere, Cerebral Infarction[Title/Abstract]) OR (Infarction, Right Hemisphere, Cerebral[Title/Abstract]) OR (Right Hemisphere, Infarction, Cerebral[Title/Abstract]) OR (Cerebral, Right Hemisphere, Infarction[Title/Abstract]) OR (Infarction, Cerebral, Right Hemisphere[Title/Abstract]) OR (Cerebral Infarction[MeSH Major Topic])

OR (Ischemic Strokes[Title/Abstract]) OR (Stroke, Ischemic[Title/Abstract]) OR (Ischaemic Stroke[Title/Abstract]) OR (Ischaemic Strokes[Title/Abstract]) OR (Stroke, Ischaemic[Title/Abstract]) OR (Cryptogenic Ischemic Stroke[Title/Abstract]) OR (Cryptogenic Ischemic Strokes[Title/Abstract]) OR (Ischemic Stroke, Cryptogenic[Title/Abstract]) OR (Stroke, Cryptogenic Ischemic[Title/Abstract]) OR (Cryptogenic Stroke[Title/Abstract]) OR (Cryptogenic Strokes[Title/Abstract]) OR (Stroke, Cryptogenic[Title/Abstract]) OR (Cryptogenic Embolism Stroke[Title/Abstract]) OR (Cryptogenic Embolism Strokes[Title/Abstract]) OR (Embolism Stroke, Cryptogenic[Title/Abstract]) OR (Stroke, Cryptogenic Embolism[Title/Abstract]) OR (Wake-up Stroke[Title/Abstract]) OR (Stroke, Wake-up[Title/Abstract]) OR (Wake up Stroke[Title/Abstract]) OR (Wake-up Strokes[Title/Abstract]) OR (Acute Ischemic Stroke[Title/Abstract]) OR (Acute Ischemic Strokes[Title/Abstract]) OR (Ischemic Stroke, Acute[Title/Abstract]) OR (Stroke, Acute Ischemic[Title/Abstract]) OR (Ischemic Stroke[MeSH Major Topic])

OR (Brain Ischemias[Title/Abstract]) OR (Ischemia, Brain[Title/Abstract]) OR (Ischemic Encephalopathy[Title/Abstract]) OR (Encephalopathy, Ischemic[Title/Abstract]) OR (Ischemic Encephalopathies[Title/Abstract])

OR (Cerebral Ischemia[Title/Abstract]) OR (Cerebral Ischemias[Title/Abstract]) OR (Ischemias, Cerebral[Title/Abstract]) OR (Ischemia, Cerebral[Title/Abstract]) OR (Brain Ischemia[MeSH Major Topic]) OR (Strokes[Title/Abstract]) OR (Cerebrovascular Accident[Title/Abstract]) OR (Cerebrovascular Accidents[Title/Abstract]) OR (CVA (Cerebrovascular Accident)[Title/Abstract]) OR (CVAs (Cerebrovascular Accident)[Title/Abstract]) OR (Cerebrovascular Apoplexy[Title/Abstract]) OR (Apoplexy, Cerebrovascular[Title/Abstract]) OR (Vascular Accident, Brain[Title/Abstract]) OR (Brain Vascular Accident[Title/Abstract]) OR (Brain Vascular Accidents[Title/Abstract]) OR (Vascular Accidents, Brain[Title/Abstract]) OR (Cerebrovascular Stroke[Title/Abstract]) OR (Cerebrovascular Strokes[Title/Abstract]) OR (Stroke, Cerebrovascular[Title/Abstract]) OR (Strokes, Cerebrovascular[Title/Abstract]) OR (Apoplexy[Title/Abstract]) OR (Cerebral Stroke[Title/Abstract]) OR (Cerebral Strokes[Title/Abstract]) OR (Stroke, Cerebral[Title/Abstract]) OR (Strokes, Cerebral[Title/Abstract]) OR (Stroke, Acute[Title/Abstract]) OR (Acute Stroke[Title/Abstract]) OR (Acute Strokes[Title/Abstract]) OR (Strokes, Acute[Title/Abstract]) OR (Cerebrovascular Accident, Acute[Title/Abstract]) OR (Acute Cerebrovascular Accident[Title/Abstract]) OR (Acute Cerebrovascular Accidents[Title/Abstract]) OR (Cerebrovascular Accidents, Acute[Title/Abstract]) OR (Stroke[MeSH Major Topic])

**AND**

(Extracellular Vesicle[Title/Abstract]) OR (Vesicle, Extracellular[Title/Abstract]) OR (Vesicles, Extracellular[Title/Abstract]) OR (Exovesicles[Title/Abstract]) OR (Exovesicle[Title/Abstract]) OR (Apoptotic Bodies[Title/Abstract]) OR (Apoptotic Body[Title/Abstract]) OR (Bodies, Apoptotic[Title/Abstract]) OR (Body, Apoptotic[Title/Abstract]) OR (Extracellular Vesicles[MeSH Major Topic])

OR (Exosomes[MeSH Major Topic]) OR (Endosome[Title/Abstract]) OR (Receptosomes[Title/Abstract]) OR (Receptosome[Title/Abstract]) OR (Endosomes[MeSH Major Topic]) OR (Cell Derived Microparticles[Title/Abstract]) OR (Cell-Derived Microparticle[Title/Abstract]) OR (Microparticle, Cell-Derived[Title/Abstract]) OR (Ectosomes[Title/Abstract]) OR (Ectosome[Title/Abstract]) OR (Microparticles, Cell-Derived[Title/Abstract]) OR (Microparticles, Cell Derived[Title/Abstract]) OR (Shedding Microvesicles[Title/Abstract]) OR (Microvesicle, Shedding[Title/Abstract]) OR (Microvesicles, Shedding[Title/Abstract]) OR (Shedding Microvesicle[Title/Abstract]) OR (Cell Membrane Microparticles[Title/Abstract]) OR (Cell Membrane Microparticle[Title/Abstract]) OR (Membrane Microparticle, Cell[Title/Abstract]) OR (Membrane Microparticles, Cell[Title/Abstract]) OR (Microparticle, Cell Membrane[Title/Abstract]) OR (Microparticles, Cell Membrane[Title/Abstract]) OR (Circulating Cell-Derived Microparticles[Title/Abstract]) OR (Cell-Derived Microparticle, Circulating[Title/Abstract]) OR (Cell-Derived Microparticles, Circulating[Title/Abstract]) OR (Circulating Cell Derived Microparticles[Title/Abstract]) OR (Circulating Cell-Derived Microparticle[Title/Abstract]) OR (Microparticle, Circulating Cell-Derived[Title/Abstract]) OR (Microparticles, Circulating Cell-Derived[Title/Abstract]) OR (Cell-Derived Microparticles[MeSH Major Topic])

**Web of Science**

**#1**

TS=(“Cerebral Infarctions” OR “Infarctions, Cerebral” OR “Infarction, Cerebral” OR “Cerebral Infarct” OR “Cerebral Infarcts” OR “Infarct, Cerebral” OR “Infarcts, Cerebral” OR “Cerebral Infarction, Left Hemisphere” OR “Left Hemisphere, Infarction, Cerebral” OR “Infarction, Left Hemisphere, Cerebral” OR “Left Hemisphere, Cerebral Infarction” OR “Cerebral, Left Hemisphere, Infarction” OR “Infarction, Cerebral, Left Hemisphere” OR “Subcortical Infarction” OR “Infarction, Subcortical” OR “Infarctions, Subcortical” OR “Subcortical Infarctions” OR “Posterior Choroidal Artery Infarction” OR “Anterior Choroidal Artery Infarction” OR “Cerebral Infarction, Right Hemisphere” OR “Right Hemisphere, Cerebral Infarction” OR “Infarction, Right Hemisphere, Cerebral” OR “Right Hemisphere, Infarction, Cerebral” OR “Cerebral, Right Hemisphere, Infarction” OR “Infarction, Cerebral, Right Hemisphere” OR “Cerebral Infarction”)

**#2**

TS=(“Ischemic Strokes” OR “Stroke, Ischemic” OR “Ischaemic Stroke” OR “Ischaemic Strokes” OR “Stroke, Ischaemic” OR “Cryptogenic Ischemic Stroke” OR “Cryptogenic Ischemic Strokes” OR “Ischemic Stroke, Cryptogenic” OR “Stroke, Cryptogenic Ischemic” OR “Cryptogenic Stroke” OR “Cryptogenic Strokes” OR “Stroke, Cryptogenic” OR “Cryptogenic Embolism Stroke” OR “Cryptogenic Embolism Strokes” OR “Embolism Stroke, Cryptogenic” OR “Stroke, Cryptogenic Embolism” OR “Wake-up Stroke” OR “Stroke, Wake-up” OR “Wake up Stroke” OR “Wake-up Strokes” OR “Acute Ischemic Stroke” OR “Acute Ischemic Strokes” OR “Ischemic Stroke, Acute” OR “Stroke, Acute Ischemic” OR “Ischemic Stroke”)

**#3**

TS=(“Brain Ischemias” OR “Ischemia, Brain” OR “Ischemic Encephalopathy” OR “Encephalopathy, Ischemic” OR “Ischemic Encephalopathies” OR “Cerebral Ischemia” OR “Cerebral Ischemias” OR “Ischemias, Cerebral” OR “Ischemia, Cerebral” OR “Brain Ischemia”)

**#4**

TS=(“Strokes” OR “Cerebrovascular Accident” OR “Cerebrovascular Accidents” OR “CVA “Cerebrovascular Accident” OR “CVAs “Cerebrovascular Accident)” OR “Cerebrovascular Apoplexy” OR “Apoplexy, Cerebrovascular” OR “Vascular Accident, Brain” OR “Brain Vascular Accident” OR “Brain Vascular Accidents” OR “Vascular Accidents, Brain” OR “Cerebrovascular Stroke” OR “Cerebrovascular Strokes” OR “Stroke, Cerebrovascular” OR “Strokes, Cerebrovascular” OR “Apoplexy” OR “Cerebral Stroke” OR “Cerebral Strokes” OR “Stroke, Cerebral” OR “Strokes, Cerebral” OR “Stroke, Acute” OR “Acute Stroke” OR “Acute Strokes” OR “Strokes, Acute” OR “Cerebrovascular Accident, Acute” OR “Acute Cerebrovascular Accident” OR “Acute Cerebrovascular Accidents” OR “Cerebrovascular Accidents, Acute” OR “Stroke”)

**#5=#1OR #2OR #3OR #4**

**#6**

TS=(“Extracellular Vesicle” OR “Vesicle, Extracellular” OR “Vesicles, Extracellular” OR “Exovesicles” OR “Exovesicle” OR “Apoptotic Bodies” OR “Apoptotic Body” OR “Bodies, Apoptotic” OR “Body, Apoptotic” OR “Extracellular Vesicles”)

**#7**

TS=(“Exosomes” OR “Endosome” OR “Receptosomes” OR “Receptosome” OR “Endosomes” OR “Cell Derived Microparticles” OR “Cell-Derived Microparticle” OR “Microparticle, Cell-Derived” OR “Ectosomes” OR “Ectosome” OR “Microparticles, Cell-Derived” OR “Microparticles, Cell Derived” OR “Shedding Microvesicles” OR “Microvesicle, Shedding” OR “Microvesicles, Shedding” OR “Shedding Microvesicle” OR “Cell Membrane Microparticles” OR “Cell Membrane Microparticle” OR “Membrane Microparticle, Cell” OR “Membrane Microparticles, Cell” OR “Microparticle, Cell Membrane” OR “Microparticles, Cell Membrane” OR “Circulating Cell-Derived Microparticles” OR “Cell-Derived Microparticle, Circulating” OR “Cell-Derived Microparticles, Circulating” OR “Circulating Cell Derived Microparticles” OR “Circulating Cell-Derived Microparticle” OR “Microparticle, Circulating Cell-Derived” OR “Microparticles, Circulating Cell-Derived” OR “Cell-Derived Microparticles”)

**#8= #6OR #7**

**#9= #5AND #8**

**Embase**

(‘Cerebral Infarctions’:ab,ti) OR (‘Infarctions, Cerebral’:ab,ti) OR (‘Infarction, Cerebral’:ab,ti) OR (‘Cerebral Infarct’:ab,ti) OR (‘Cerebral Infarcts’:ab,ti) OR (‘Infarct, Cerebral’:ab,ti) OR (‘Infarcts, Cerebral’:ab,ti) OR (‘Cerebral Infarction, Left Hemisphere’:ab,ti) OR (‘Left Hemisphere, Infarction, Cerebral’:ab,ti) OR (‘Infarction, Left Hemisphere, Cerebral’:ab,ti) OR (‘Left Hemisphere, Cerebral Infarction’:ab,ti) OR (‘Cerebral, Left Hemisphere, Infarction’:ab,ti) OR (‘Infarction, Cerebral, Left Hemisphere’:ab,ti) OR (‘Subcortical Infarction’:ab,ti) OR (‘Infarction, Subcortical’:ab,ti) OR (‘Infarctions, Subcortical’:ab,ti) OR (‘Subcortical Infarctions’:ab,ti) OR (‘Posterior Choroidal Artery Infarction’:ab,ti) OR (‘Anterior Choroidal Artery Infarction’:ab,ti) OR (‘Cerebral Infarction, Right Hemisphere’:ab,ti) OR (‘Right Hemisphere, Cerebral Infarction’:ab,ti) OR (‘Infarction, Right Hemisphere, Cerebral’:ab,ti) OR (‘Right Hemisphere, Infarction, Cerebral’:ab,ti) OR (‘Cerebral, Right Hemisphere, Infarction’:ab,ti) OR (‘Infarction, Cerebral, Right Hemisphere’:ab,ti) OR (‘Cerebral Infarction’/exp)

OR (‘Ischemic Strokes’:ab,ti) OR (‘Stroke, Ischemic’:ab,ti) OR (‘Ischaemic Stroke’:ab,ti) OR (‘Ischaemic Strokes’:ab,ti) OR (‘Stroke, Ischaemic’:ab,ti) OR (‘Cryptogenic Ischemic Stroke’:ab,ti) OR (‘Cryptogenic Ischemic Strokes’:ab,ti) OR (‘Ischemic Stroke, Cryptogenic’:ab,ti) OR (‘Stroke, Cryptogenic Ischemic’:ab,ti) OR (‘Cryptogenic Stroke’:ab,ti) OR (‘Cryptogenic Strokes’:ab,ti) OR (‘Stroke, Cryptogenic’:ab,ti) OR (‘Cryptogenic Embolism Stroke’:ab,ti) OR (‘Cryptogenic Embolism Strokes’:ab,ti) OR (‘Embolism Stroke, Cryptogenic’:ab,ti) OR (‘Stroke, Cryptogenic Embolism’:ab,ti) OR (‘Wake-up Stroke’:ab,ti) OR (‘Stroke, Wake-up’:ab,ti) OR (‘Wake up Stroke’:ab,ti) OR (‘Wake-up Strokes’:ab,ti) OR (‘Acute Ischemic Stroke’:ab,ti) OR (‘Acute Ischemic Strokes’:ab,ti) OR (‘Ischemic Stroke, Acute’:ab,ti) OR (‘Stroke, Acute Ischemic’:ab,ti) OR (‘Ischemic Stroke’/exp)OR (‘Brain Ischemias’:ab,ti) OR (‘Ischemia, Brain’:ab,ti) OR (‘Ischemic Encephalopathy’:ab,ti) OR (‘Encephalopathy, Ischemic’:ab,ti) OR (‘Ischemic Encephalopathies’:ab,ti) OR (‘Cerebral Ischemia’:ab,ti) OR (‘Cerebral Ischemias’:ab,ti) OR (‘Ischemias, Cerebral’:ab,ti) OR (‘Ischemia, Cerebral’:ab,ti) OR (‘Brain Ischemia’/exp) OR (‘Strokes’:ab,ti) OR (‘Cerebrovascular Accident’:ab,ti) OR (‘Cerebrovascular Accidents’:ab,ti) OR (‘CVA (Cerebrovascular Accident)’:ab,ti) OR (‘CVAs (Cerebrovascular Accident)’:ab,ti) OR (‘Cerebrovascular Apoplexy’:ab,ti) OR (‘Apoplexy, Cerebrovascular’:ab,ti) OR (‘Vascular Accident, Brain’:ab,ti) OR (‘Brain Vascular Accident’:ab,ti) OR (‘Brain Vascular Accidents’:ab,ti) OR (‘Vascular Accidents, Brain’:ab,ti) OR (‘Cerebrovascular Stroke’:ab,ti) OR (‘Cerebrovascular Strokes’:ab,ti) OR (‘Stroke, Cerebrovascular’:ab,ti) OR (‘Strokes, Cerebrovascular’:ab,ti) OR (‘Apoplexy’:ab,ti) OR (‘Cerebral Stroke’:ab,ti) OR (‘Cerebral Strokes’:ab,ti) OR (‘Stroke, Cerebral’:ab,ti) OR (‘Strokes, Cerebral’:ab,ti) OR (‘Stroke, Acute’:ab,ti) OR (‘Acute Stroke’:ab,ti) OR (‘Acute Strokes’:ab,ti) OR (‘Strokes, Acute’:ab,ti) OR (‘Cerebrovascular Accident, Acute’:ab,ti) OR (‘Acute Cerebrovascular Accident’:ab,ti) OR (‘Acute Cerebrovascular Accidents’:ab,ti) OR (‘Cerebrovascular Accidents, Acute’:ab,ti) OR (‘Stroke’/exp)

**AND**

(‘Extracellular Vesicle’:ab,ti) OR (‘Vesicle, Extracellular’:ab,ti) OR (‘Vesicles, Extracellular’:ab,ti) OR (‘Exovesicles’:ab,ti) OR (‘Exovesicle’:ab,ti) OR (‘Apoptotic Bodies’:ab,ti) OR (‘Apoptotic Body’:ab,ti) OR (‘Bodies, Apoptotic’:ab,ti) OR (‘Body, Apoptotic’:ab,ti) OR (‘Extracellular Vesicles’/exp) OR (‘Exosomes’/exp) OR (‘Endosome’:ab,ti) OR (‘Receptosomes’:ab,ti) OR (‘Receptosome’:ab,ti) OR (‘Endosomes’/exp) OR (‘Cell Derived Microparticles’:ab,ti) OR (‘Cell-Derived Microparticle’:ab,ti) OR (‘Microparticle, Cell-Derived’:ab,ti) OR (‘Ectosomes’:ab,ti) OR (‘Ectosome’:ab,ti) OR (‘Microparticles, Cell-Derived’:ab,ti) OR (‘Microparticles, Cell Derived’:ab,ti) OR (‘Shedding Microvesicles’:ab,ti) OR (‘Microvesicle, Shedding’:ab,ti) OR (‘Microvesicles, Shedding’:ab,ti) OR (‘Shedding Microvesicle’:ab,ti) OR (‘Cell Membrane Microparticles’:ab,ti) OR (‘Cell Membrane Microparticle’:ab,ti) OR (‘Membrane Microparticle, Cell’:ab,ti) OR (‘Membrane Microparticles, Cell’:ab,ti) OR (‘Microparticle, Cell Membrane’:ab,ti) OR (‘Microparticles, Cell Membrane’:ab,ti) OR (‘Circulating Cell-Derived Microparticles’:ab,ti) OR (‘Cell-Derived Microparticle, Circulating’:ab,ti) OR (‘Cell-Derived Microparticles, Circulating’:ab,ti) OR (‘Circulating Cell Derived Microparticles’:ab,ti) OR (‘Circulating Cell-Derived Microparticle’:ab,ti) OR (‘Microparticle, Circulating Cell-Derived’:ab,ti) OR (‘Microparticles, Circulating Cell-Derived’:ab,ti) OR (‘Cell-Derived Microparticles’/exp)

**Fig. S1. Forest plot summarizing the relationship between SC-EVs species (i.e. allogeneic, xenogenic, and syngeneic) and infarct volume in preclinical models of [cerebral](../../../../D:/%25E8%25BD%25AF%25E4%25BB%25B6/360%25E6%25B8%25B8%25E8%25A7%2588%25E5%2599%25A8/Youdao/Dict/8.10.3.0/resultui/html/index.html#/javascript:;) [infarction](../../../../D:/%25E8%25BD%25AF%25E4%25BB%25B6/360%25E6%25B8%25B8%25E8%25A7%2588%25E5%2599%25A8/Youdao/Dict/8.10.3.0/resultui/html/index.html#/javascript:;).**

**
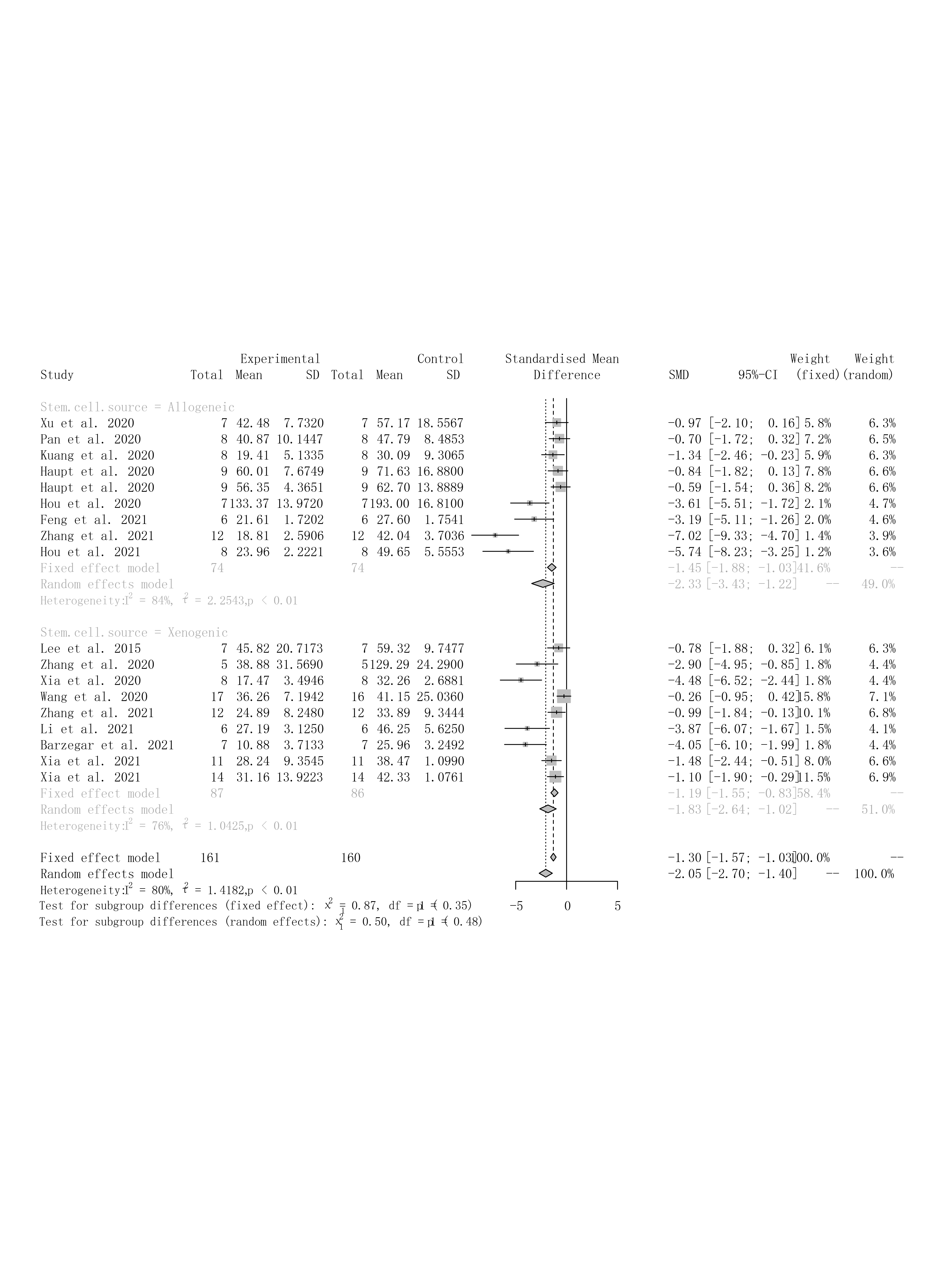
**

**Fig.S2. Forest plot summarizing the relationship between SC-EVs-treated animal model species (rat versus mouse) and infarct volume in preclinical models of [cerebral](../../../../D:/%25E8%25BD%25AF%25E4%25BB%25B6/360%25E6%25B8%25B8%25E8%25A7%2588%25E5%2599%25A8/Youdao/Dict/8.10.3.0/resultui/html/index.html#/javascript:;) [infarction](../../../../D:/%25E8%25BD%25AF%25E4%25BB%25B6/360%25E6%25B8%25B8%25E8%25A7%2588%25E5%2599%25A8/Youdao/Dict/8.10.3.0/resultui/html/index.html#/javascript:;).**

**
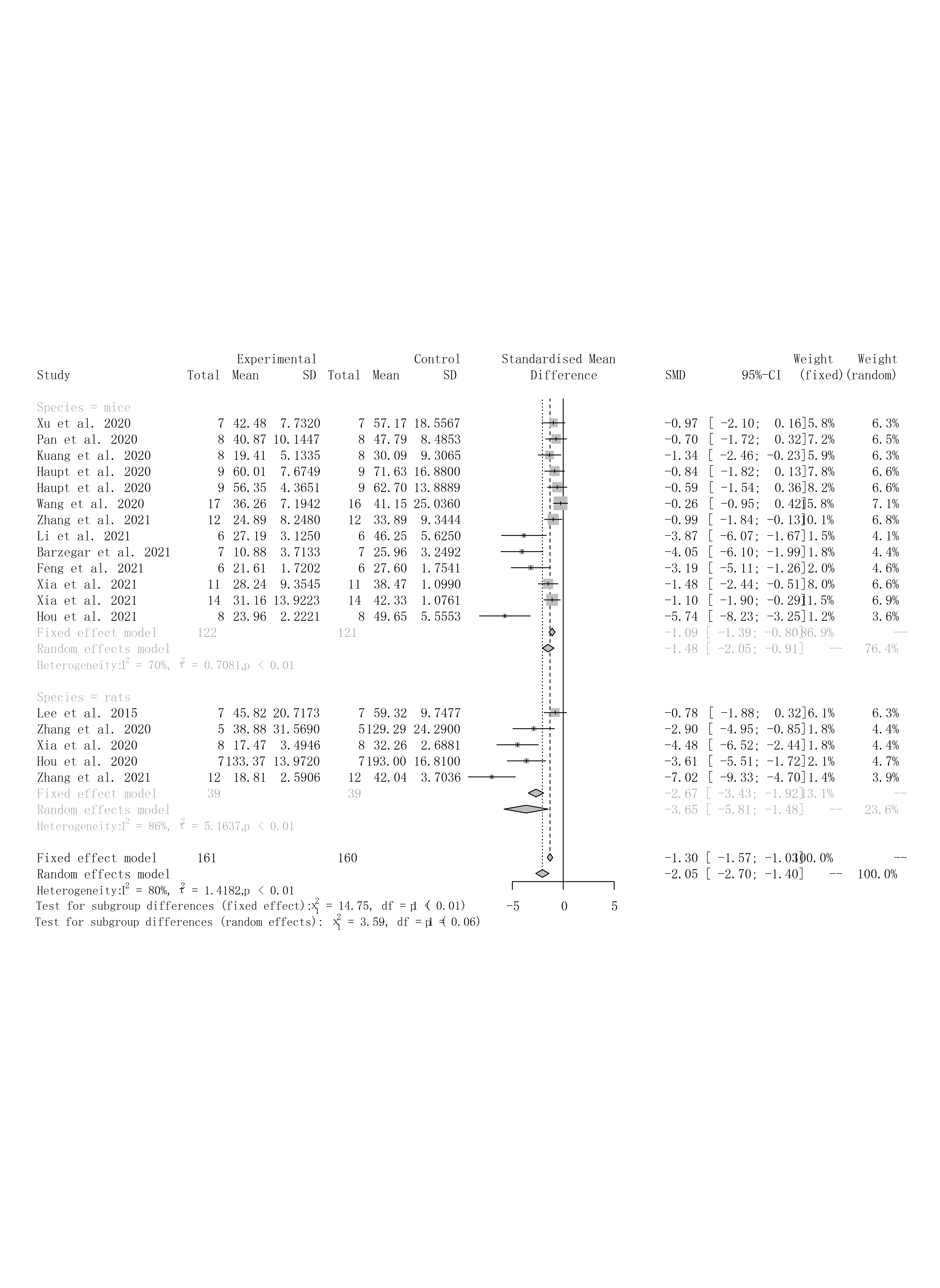
**

**Fig.S3. Forest plot summarizing the relationship between preclinical models of [cerebral](../../../../D:/%25E8%25BD%25AF%25E4%25BB%25B6/360%25E6%25B8%25B8%25E8%25A7%2588%25E5%2599%25A8/Youdao/Dict/8.10.3.0/resultui/html/index.html#/javascript:;) [infarction](../../../../D:/%25E8%25BD%25AF%25E4%25BB%25B6/360%25E6%25B8%25B8%25E8%25A7%2588%25E5%2599%25A8/Youdao/Dict/8.10.3.0/resultui/html/index.html#/javascript:;) and infarct volume in preclinical models.**

**
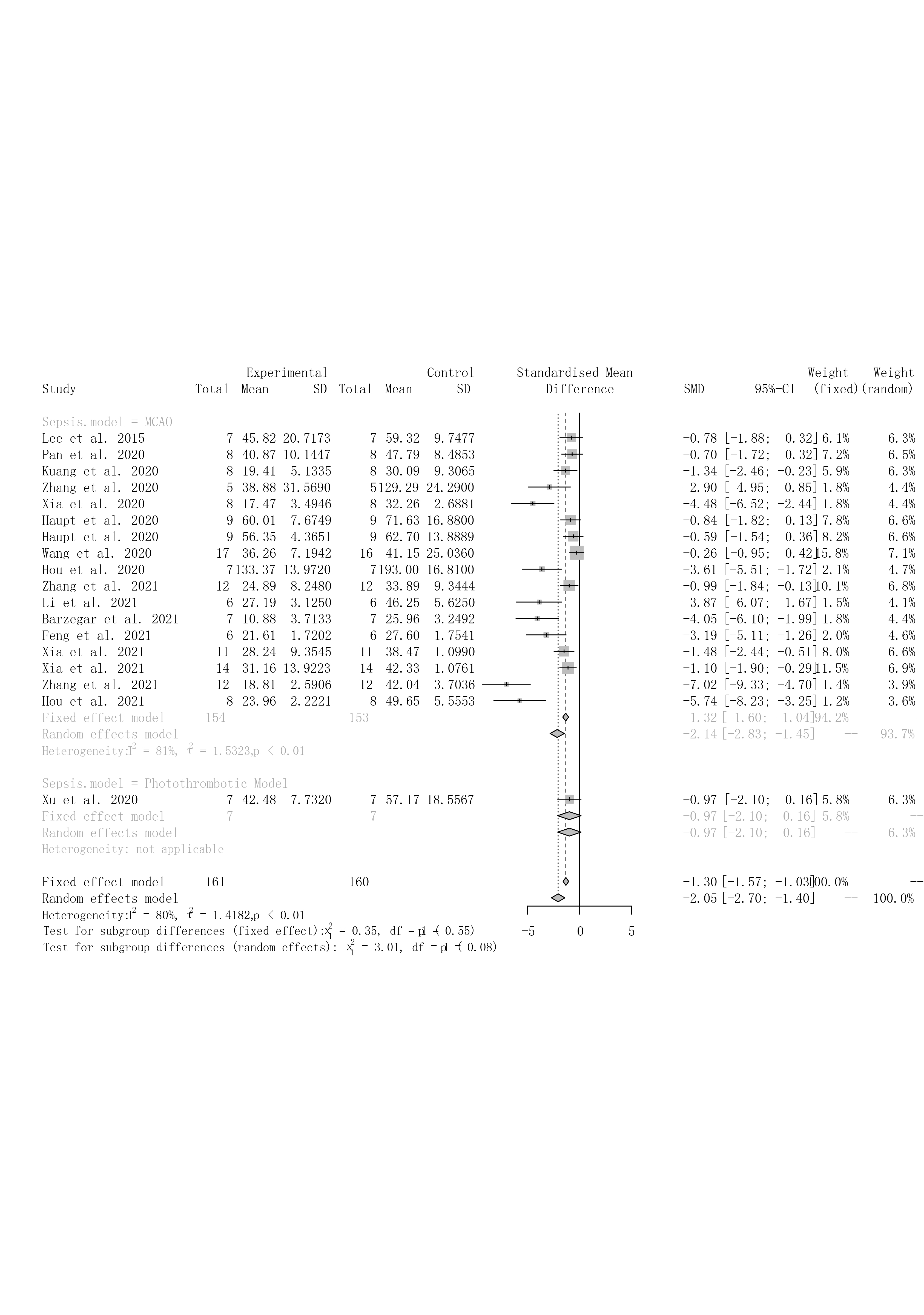
**

**Fig.S4. Forest plot summarizing the relationship between SC-EVs types and infarct volume in preclinical models of [cerebral](../../../../D:/%25E8%25BD%25AF%25E4%25BB%25B6/360%25E6%25B8%25B8%25E8%25A7%2588%25E5%2599%25A8/Youdao/Dict/8.10.3.0/resultui/html/index.html#/javascript:;) [infarction](../../../../D:/%25E8%25BD%25AF%25E4%25BB%25B6/360%25E6%25B8%25B8%25E8%25A7%2588%25E5%2599%25A8/Youdao/Dict/8.10.3.0/resultui/html/index.html#/javascript:;).**

**
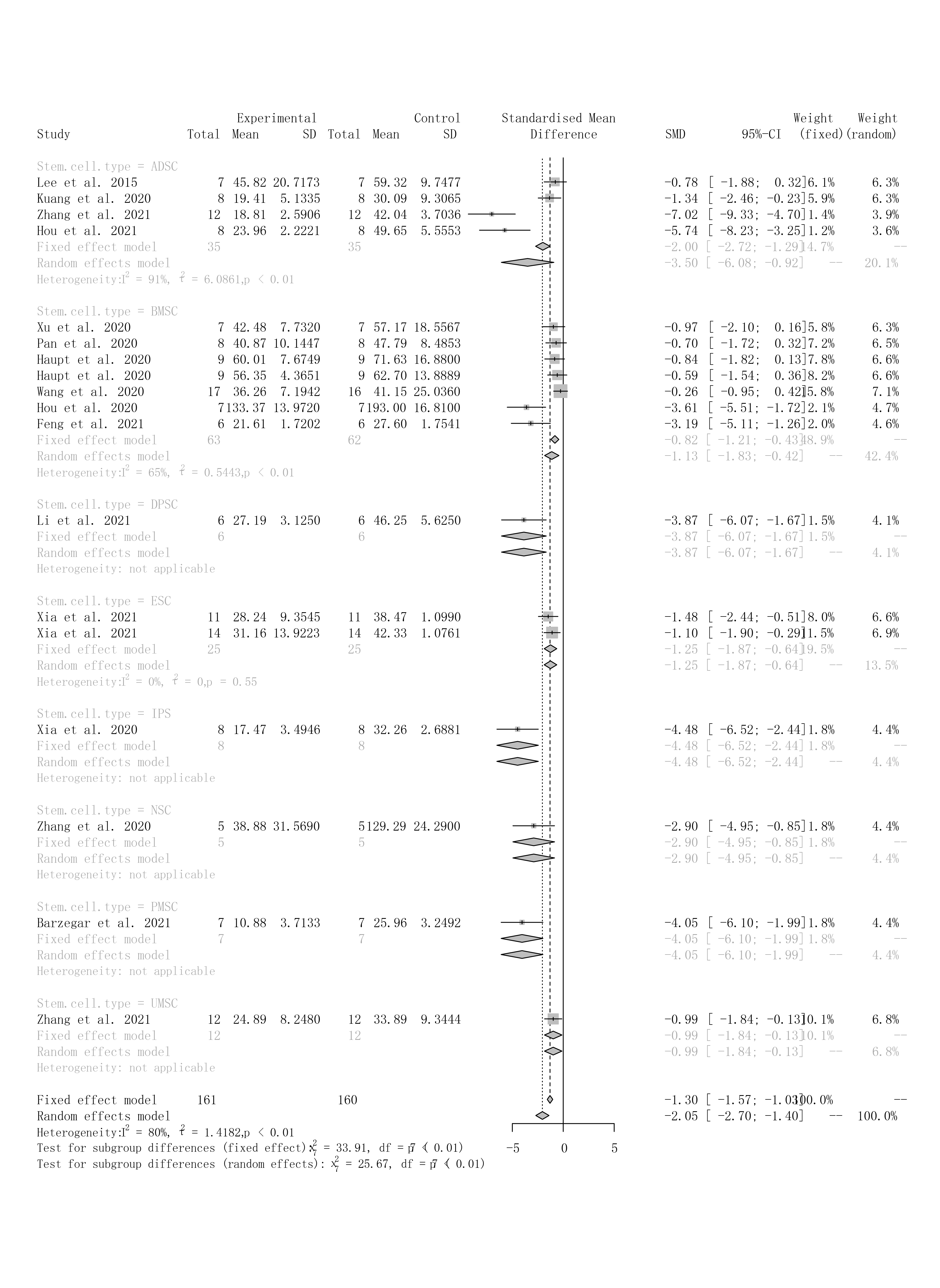
**

**Fig.S5. Forest plot summarizing the relationship between animal sex and infarct volume in preclinical models of [cerebral](../../../../D:/%25E8%25BD%25AF%25E4%25BB%25B6/360%25E6%25B8%25B8%25E8%25A7%2588%25E5%2599%25A8/Youdao/Dict/8.10.3.0/resultui/html/index.html#/javascript:;) [infarction](../../../../D:/%25E8%25BD%25AF%25E4%25BB%25B6/360%25E6%25B8%25B8%25E8%25A7%2588%25E5%2599%25A8/Youdao/Dict/8.10.3.0/resultui/html/index.html#/javascript:;).**

**
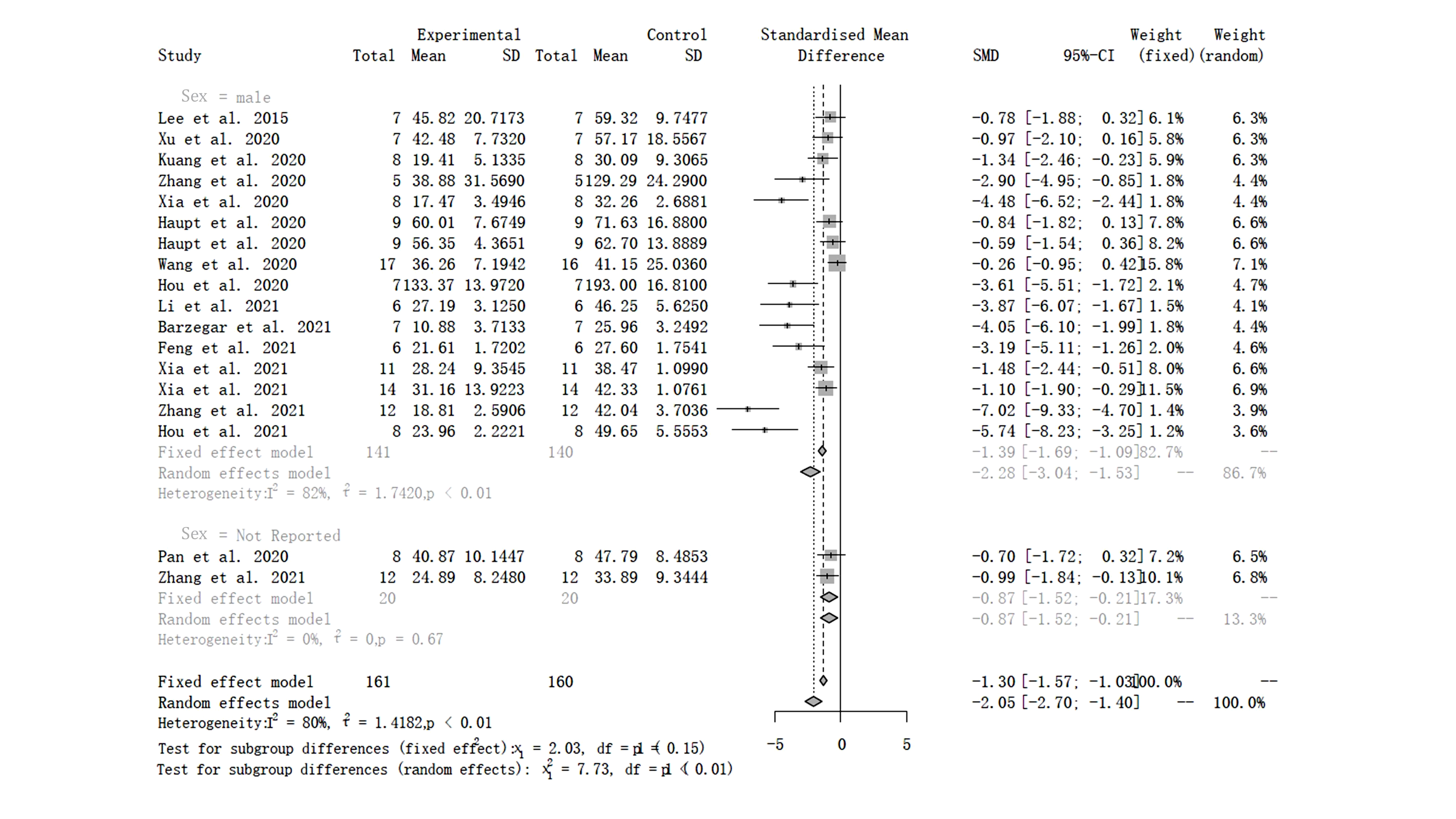
**

**Fig.S6. Forest plot summarizing the relationship between SC-EVs administration route and infarct volume in preclinical models of [cerebral](../../../../D:/%25E8%25BD%25AF%25E4%25BB%25B6/360%25E6%25B8%25B8%25E8%25A7%2588%25E5%2599%25A8/Youdao/Dict/8.10.3.0/resultui/html/index.html#/javascript:;) [infarction](../../../../D:/%25E8%25BD%25AF%25E4%25BB%25B6/360%25E6%25B8%25B8%25E8%25A7%2588%25E5%2599%25A8/Youdao/Dict/8.10.3.0/resultui/html/index.html#/javascript:;).**

**
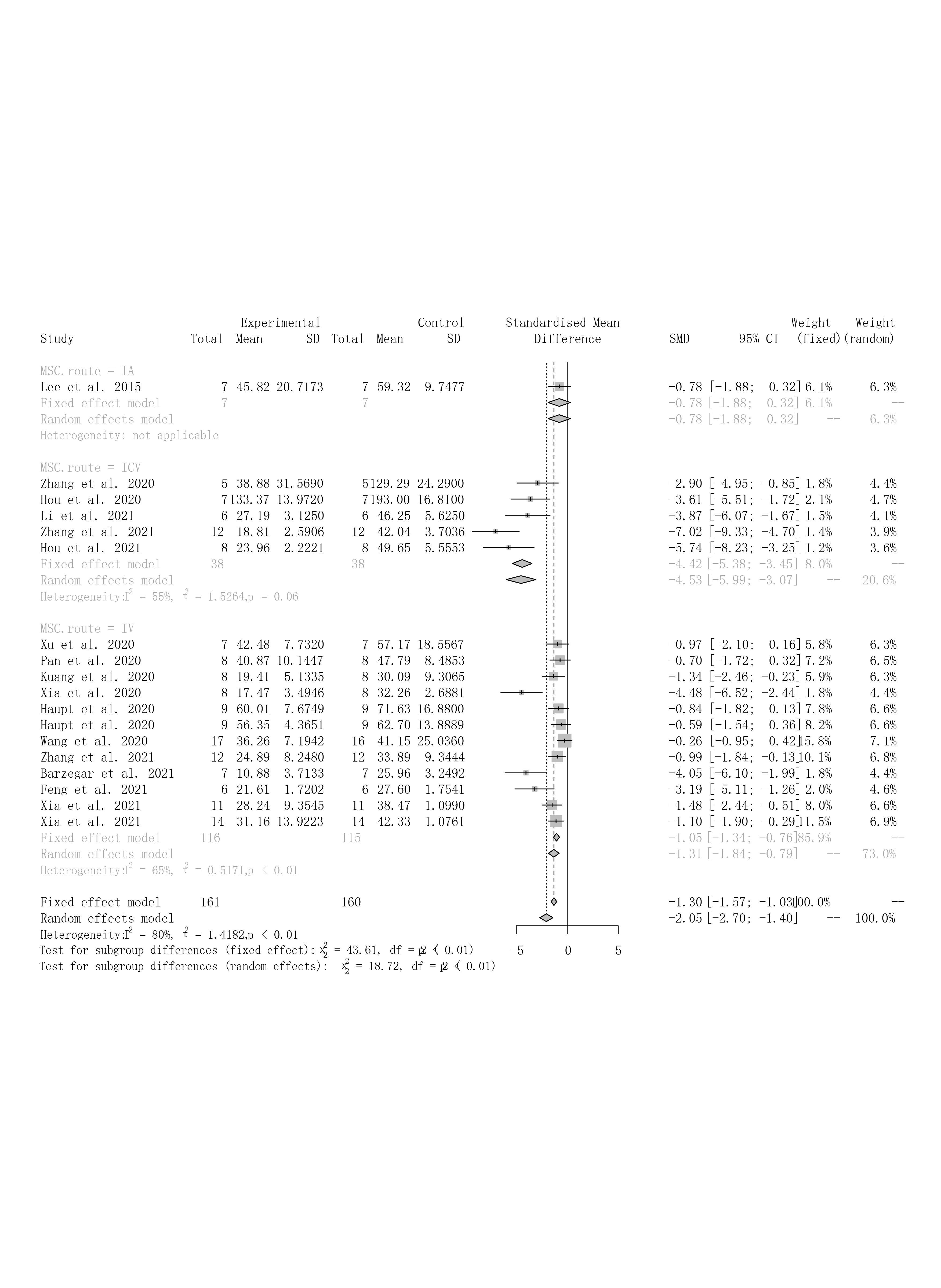
**

**Fig.S7. Forest plot summarizing the relationship between therapy timing of administration and infarct volume in preclinical models of [cerebral](../../../../D:/%25E8%25BD%25AF%25E4%25BB%25B6/360%25E6%25B8%25B8%25E8%25A7%2588%25E5%2599%25A8/Youdao/Dict/8.10.3.0/resultui/html/index.html#/javascript:;) [infarction](../../../../D:/%25E8%25BD%25AF%25E4%25BB%25B6/360%25E6%25B8%25B8%25E8%25A7%2588%25E5%2599%25A8/Youdao/Dict/8.10.3.0/resultui/html/index.html#/javascript:;).**

**
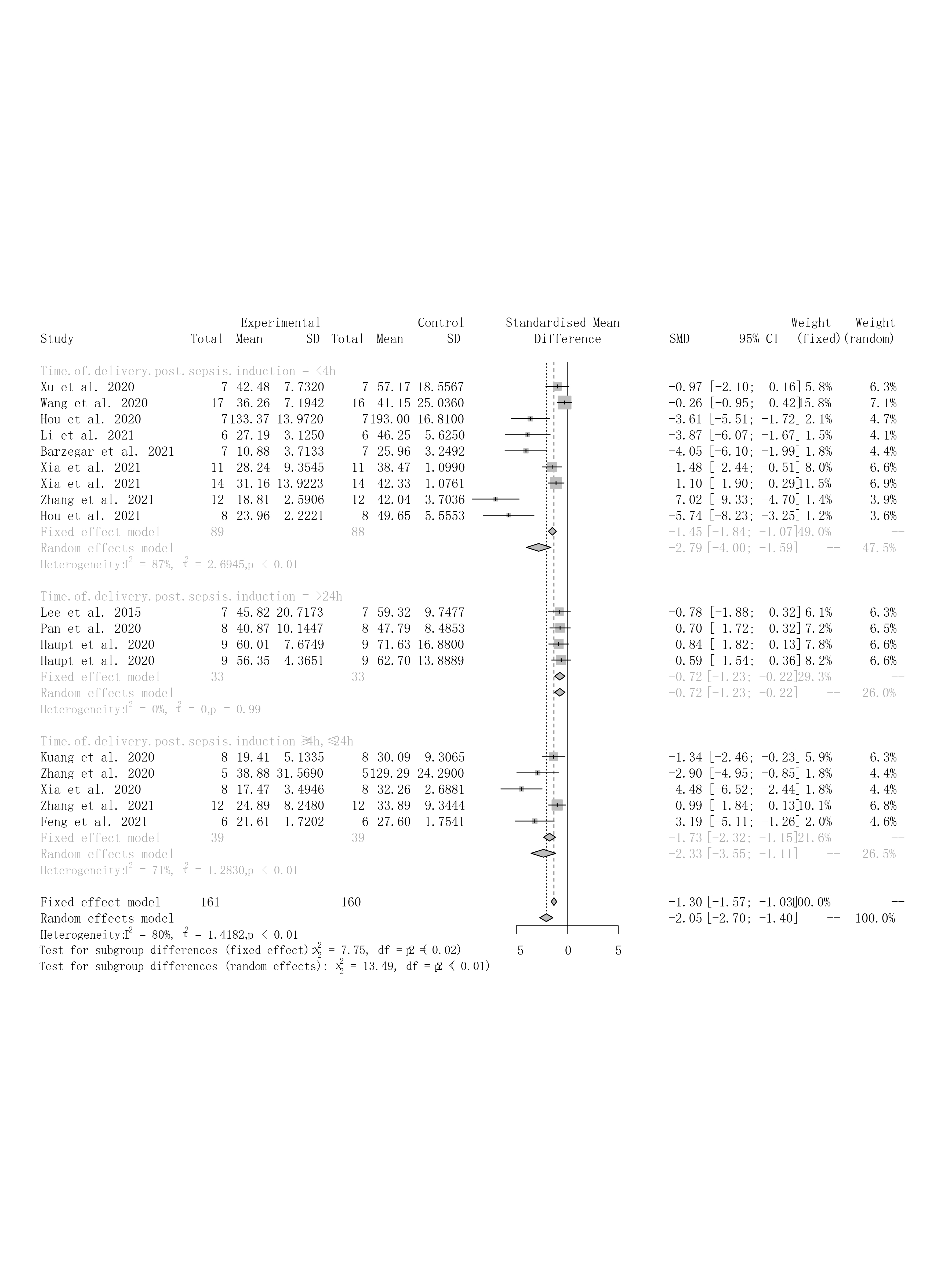
**

**Fig.S8. Forest plot summarizing the relationship between extraction method of EVs and infarct volume in preclinical models of [cerebral](../../../../D:/%25E8%25BD%25AF%25E4%25BB%25B6/360%25E6%25B8%25B8%25E8%25A7%2588%25E5%2599%25A8/Youdao/Dict/8.10.3.0/resultui/html/index.html#/javascript:;) [infarction](../../../../D:/%25E8%25BD%25AF%25E4%25BB%25B6/360%25E6%25B8%25B8%25E8%25A7%2588%25E5%2599%25A8/Youdao/Dict/8.10.3.0/resultui/html/index.html#/javascript:;).**

**
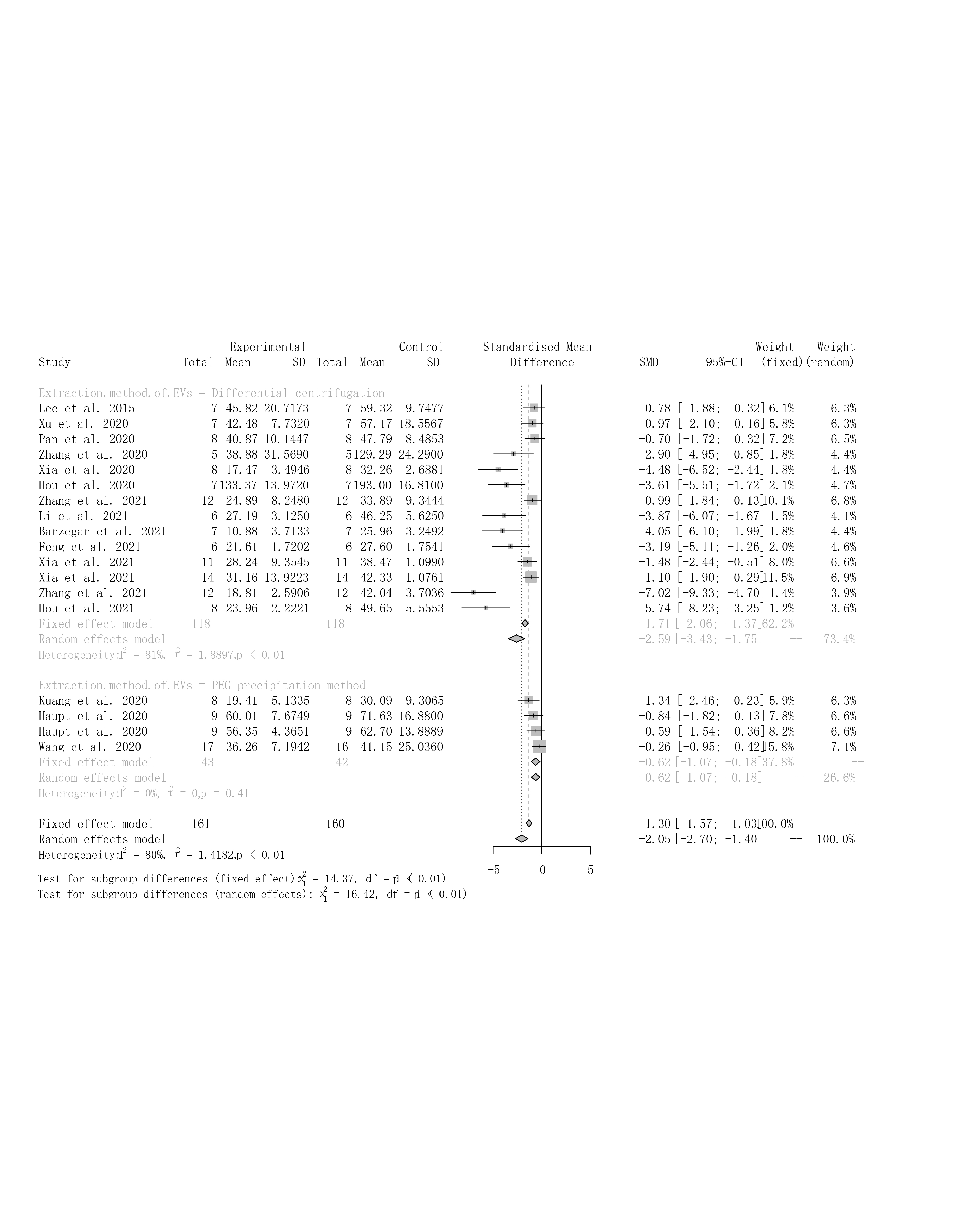
**
